# Hydrogels Incorporating Donor–Acceptor Stenhouse Adducts as a Platform for Photoinduced, On‐Off Switchable Release of Small Molecule Cargos

**DOI:** 10.1002/marc.202500868

**Published:** 2026-01-31

**Authors:** Tristan N. Dell, Ana Cammack‐Najera, Rea Tresa, Farzina Matubbar, Beyzanur Kaya, Uthaya Lathan, Mohamed Chami, Ray G. DiNardi, Omar Rifaie‐Graham, Jonathan P. Wojciechowski, Molly M. Stevens

**Affiliations:** ^1^ Department of Materials Department of Bioengineering and Institute of Biomedical Engineering Imperial College London London UK; ^2^ Department of Physiology Anatomy and Genetics Department of Engineering Science Kavli Institute for Nanoscience Discovery University of Oxford Oxford UK; ^3^ Department of Chemistry School of Physical and Chemical Sciences Queen Mary University of London London UK; ^4^ BioEM Lab Biozentrum University of Basel Basel Switzerland

**Keywords:** Donor–acceptor Stenhouse adducts, Drug release, Hydrogels, Photoswitches, Polymersomes

## Abstract

Modulating biomaterial properties using light holds great promise for biomedical applications, such as drug delivery, as it is non‐invasive and offers both spatial and temporal control. Visible light is particularly salient for stimulation of cell‐interfacing materials, as it is cyto‐compatible; however, this limits the number of photoswitches appropriate for these applications. In this work, we use donor–acceptor Stenhouse adduct (DASA) functionalized polymers comprising poly(ethylene glycol)‐*b‐*poly(hexyl methacrylate) to make visible light‐responsive polymersomes, and use these to encapsulate a model drug cargo. We demonstrate that release of the model cargo can be triggered using visible light when the polymersomes are loaded into poly(ethylene glycol) hydrogels. Moreover, ON/OFF switchable cargo release was demonstrated by modulating the light stimulation of the hydrogel. We envisage this could be used to dynamically modulate hydrogel properties in clinically relevant applications for controlled delivery of small molecule therapeutic agents, such as advanced in vitro tissue models and implantable drug‐eluting scaffolds.

## Graphical abstract

Donor–acceptor Stenhouse aDdducts (DASAs) are known for their photoinduced tautomerization, which, when conjugated to block copolymers, can self‐assemble into tuneable polymersomes. These DASA‐polymersomes are being investigated for light‐controlled cargo release in biomedical applications. This work shows light‐controlled release of a fluorescent cargo from polymersomes encapsulated in a hydrogel scaffold.

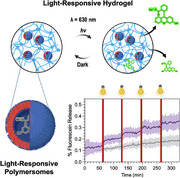

## 1 Introduction

Stimuli‐responsive hydrogels have distinct advantages compared to conventional diffusion‐based systems because the release is controlled using an external stimulus. This has broad potential in applications such as cell culture to create gradients of chemokines that influence cell behavior, implantable soft robots that respond to a local environment depending on the stimulus applied (e.g., light, temperature, pH), or drug release that can be controlled with respect to the location and timing requirements [1, 2, 3, 4]. Additionally, hydrogels can be composed of biocompatible materials that can be tailored to match the physical and mechanical properties of their environment [5, 6, 7]. Light, temperature, pH, and enzymatic activity can all be used as stimuli to control drug release. However, light offers additional advantages over other stimuli as it is non‐invasive and offers spatiotemporal control.

Early examples of light‐triggered drug release from hydrogels utilized the photoisomerization of azobenzene‐functionalized poly(*N*‐isopropylacrylamide) (PNIPAam), where excitation of the azobenzene to the *cis* isomer gave a small, but measurable difference in the release of model small molecules [8]. Incorporating photoswitchable functional groups into amphiphiles that form supramolecular hydrogels demonstrated light‐triggered cargo release enabled by on‐demand dissolution of the hydrogels. Whereas tethering the cargo via a photo‐cleavable moiety (i.e., *o*‐nitrobenzyl derivative) could eliminate any residual release based on diffusion in the absence of light [9]. Chemically tethering cargo gives exceptional control over release; however, requires suitable functional groups to attach to a hydrogel and typically several synthetic steps to synthesize [10]. An alternative strategy is to use stimuli‐responsive nanoparticles, such as polymersomes, liposomes, micelles, or peptide ensembles, for cargo encapsulation [11, 12, 13]. This approach enables tailoring of the encapsulated payload to specific applications, while harnessing the stimuli‐responsive properties of a nanoparticle to achieve controlled release.

Photoswitches such as azobenzenes, spiropyrans, and donor–acceptor Stenhouse adducts (DASAs) have been used as the functional moieties in light‐responsive nanoparticles. Many azobenzene‐based systems are isomerized using UV‐light as a trigger [14, 15], limiting cytocompatibility and creating challenges due to the low tissue penetration depth of UV light. There have been more recent efforts to modify azobenzenes, shifting their switching wavelength into the visible or NIR spectrum [16, 17, 18], or using upconversion nanoparticles (UCNPs) to access physiologically relevant wavelengths of light to trigger release [19, 20]. Similarly, spiropyrans have been used in light‐responsive nanoparticles [21, 22, 23]. However, spiropyrans usually need irradiation by two different wavelengths to switch reversibly between merocyanine and spiropyran isomers [24], and accessing switching wavelengths in the NIR range has remained elusive. Furthermore, spiropyrans have relatively poor switching recovery in aqueous environments, as the merocyanine isomer has a tendency to form H‐aggregates and J‐aggregates [24] and is prone to photodegradation and hydrolysis [25]. These factors limit their applicability in many biomedical contexts. Other NIR‐sensitive systems, such as photothermal NIR dyes [26], metal‐organic frameworks (MOFs) [27, 28], mesoporous nanoparticles [28], and plasmonic gold nanoparticles [29, 30], show promise in cargo release at physiologically relevant wavelengths, but often have issues with cargo leaching, as the payloads are usually not coupled covalently.

Donor–acceptor Stenhouse adducts (DASAs) present desirable photoswitching properties as they are responsive to a range of wavelengths within the visible spectrum, are negatively photochromic, and can be switched reversibly over multiple cycles [31, 32, 33, 34, 35]. Moreover, DASAs are classified as negative photoswitches (i.e., they lose coloration upon irradiation with light), allowing for greater light penetration throughout the photoswitching process due to decreasing competition with neighboring DASAs for absorption of visible light. DASAs have been incorporated into organogels as pendant groups through direct conjugation of the DASA into the network architecture, directly into the polymer backbone, or via post‐modification of the network after crosslinking [36, 37, 38, 39, 40]. However, these examples are incompatible with aqueous environments, a key requirement to use these systems in biomedical applications [36]. Incorporation of the DASAs into self‐assembled structures has been demonstrated as an important strategy for ‘shielding’ DASAs from unwanted interactions with polar solvent molecules [35, 36, 37, 38, 39, 40].

**FIGURE 1:**
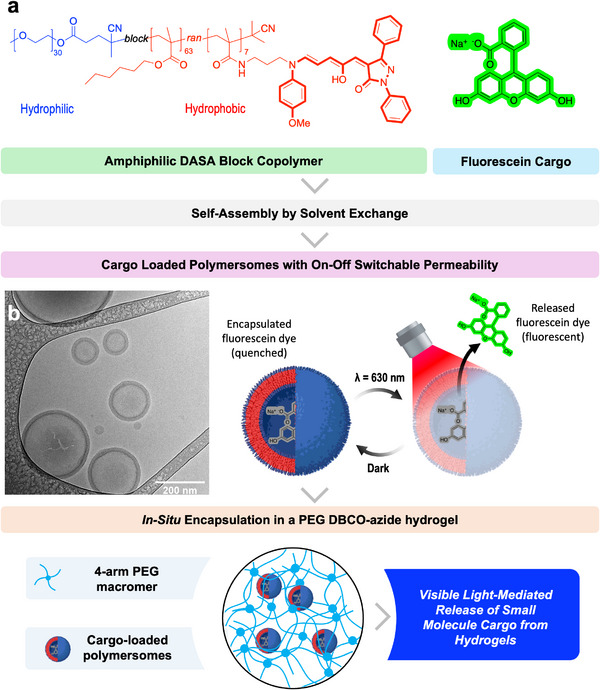
Schematic overview of the constituent parts of the system. (A) Structure of DASA block copolymers and schematic of polymersome self‐assembly and subsequent loading into PEG hydrogels crosslinked via strain‐promoted azide‐alkyne cycloaddition, click chemistry. (B, inset) Cryo‐TEM of empty DASA‐polymersomes.

In this work, self‐assembled structures were used to shield the DASA moieties from water, allowing for reversible photo‐switching in aqueous conditions. This enabled the preservation of DASA photoswitching in hydrogels, as described herein. Building on previous work demonstrating DASA‐functionalized polymersomes as vehicles for light‐triggered release of small‐molecule drug models [41], we encapsulate these nanoparticles within hydrogels to create depots for light‐controlled, active cargo delivery (Figure 1). We demonstrate photoactive hydrogels that derive their light‐responsive properties from DASA polymersomes embedded within the polymer network (Figure 1). These hydrogels were fabricated with the DASA polymersomes in situ, generating a polymer network to entrap the DASA‐functionalized nanoparticles, thus conveying their photoactive properties to the bulk structure. Using this system, we show that DASA polymersomes that are physically retained in the mesh of PEG dibenzocyclooctyne (DBCO)‐azide hydrogels can be employed for controlled light‐modulated release of a model cargo in localized aqueous environments in an ON/OFF manner.

## 2 Results and Discussion

**FIGURE 2:**
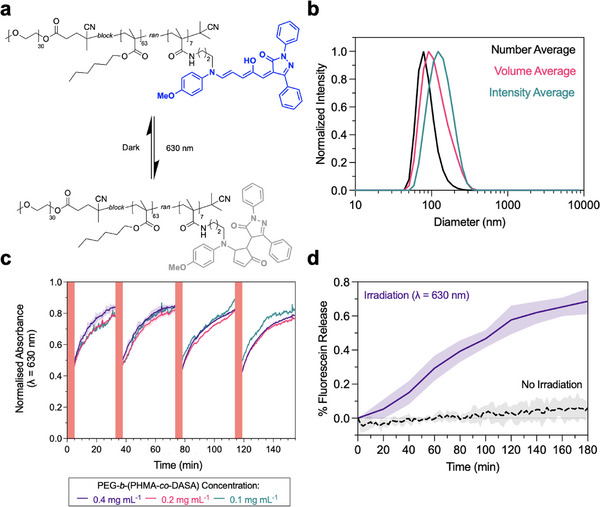
Characterization of DASA polymersomes in aqueous dispersion. (a) Structure of the amphiphilic block copolymer, PEG‐*b*‐(PHMA‐*co*‐DASA), used to prepare DASA polymersomes, showing both the linear triene‐enol (open, dark) conformation, and the cyclic cyclopentenone (closed, irradiated) conformation. (b) DLS measurement of fluorescein‐encapsulating DASA polymersomes formed by solvent exchange and purification by dialysis. (c) UV–vis absorbance spectroscopy data, showing reversible DASA switching of DASA polymersomes in PBS. Samples were irradiated for 5 min (λ = 630 nm, 5.4 mW cm^−2^), then recovery from the cyclopentenone form to the triene‐enol form was monitored by UV–vis over 30 min in the dark. Recovery was measured by tracking the intensity of the absorbance peak of the triene‐enol DASA conformation at λ = 630 nm. This was repeated over four cycles. (n = 3, SD shown). (d) Fluorescence spectroscopy was used to monitor the release of fluorescein from DASA polymersomes in an aqueous dispersion. The aqueous polymersome dispersion was irradiated continuously (λ = 630 nm), monitoring fluorescence at 20 min intervals. This was compared to a control, which was kept in the dark and baseline corrected (N = 3, SD shown). This gave a maximum concentration of fluorescein loaded in the particles of 25.9 µM, corresponding to an encapsulation efficiency of 0.1%. For quantification as concentration (nM), see Figure S8.

We synthesized poly(ethylene glycol)‐*b*‐(poly(hexamethyl acrylate)‐*co*‐(DASA)) (PEG‐*b*‐(PHMA‐*co*‐DASA)), an amphiphilic block copolymer with a pyrazalone‐based DASA copolymerized into the hydrophobic block by modification of a previously described method from Rifaie–Graham et al. (Figures S1–S4) [41]. Briefly, chain extension of a PEG chain transfer agent with hexyl methacrylate (HMA) and pentafluorophenyl methacrylate (PFPMA) by Reversible Addition Fragmentation chain‐Transfer (RAFT) radical polymerization yielded an amphiphilic diblock copolymer with randomly distributed activated ester motifs in the hydrophobic block PEG‐*b*‐PPFPMA‐*co*‐PHMA. The polymer was then reacted with *N*‐(4‐methoxyphenyl)propane‐1,3‐diamine, yielding an aromatic amine pendant group that acted as a precursor to the formation of DASAs via reaction with a pyrazolone‐based furan adduct, generating the photoswitchable polymer PEG‐*b‐*(PHMA‐*co*‐DASA). Polymersomes were formed via dropwise addition of PEG‐*b*‐(PHMA‐*co*‐DASA) in ultrapure water, followed by dialysis (MWCO = 1 kDa). Morphological analysis by cryogenic transmission electron microscopy (cryo‐TEM) revealed the formation of polymersomes, as characterized by a spherical morphology featuring a bilayer around a central aqueous lumen (Figure 1b). Dynamic light scattering (DLS) measurements of the fluorescein encapsulating polymersomes in ultrapure water showed particle diameters, *D_h_* = 123.9 ± 2.2 nm (Figure 2b). DLS measurements for empty polymersomes were also collected (Figure S5).

Before entrapping the polymersomes in a hydrogel mesh, we investigated reversible photoswitching of the DASA polymersomes in ultrapure water (Figure 2c). The DASA polymersomes were irradiated for 5 min (λ = 630 nm), then allowed to thermally revert at room temperature (20°C) to the open, triene‐enol for 30 min in the absence of light. This process was repeated four times, demonstrating cycle‐to‐cycle recovery between the open and closed forms of the DASA. Good reproducibility was also shown over a range of concentrations: 0.1, 0.2, and 0.4 mg mL^−1^.

Having demonstrated reversible switching of the DASA polymersomes in aqueous conditions, the light‐triggered release of the fluorescein cargo was investigated (Figure 2d; Figure S6). It has been previously reported that upon irradiation, the membrane of DASA polymersomes can switch from a non‐permeable to a permeable state, allowing for the release of encapsulated cargo [41, 42]. Aromatic fluorescent dyes exhibit concentration‐dependent fluorescence quenching, allowing for convenient monitoring of cargo release using fluorescence spectroscopy [43, 44]. Herein, sodium fluorescein was encapsulated at self‐quenching concentrations as a model small molecule drug cargo, followed by purification using dialysis (MWCO = 1 kDa) to remove most of the unencapsulated dye. Rhodamine B and eosin Y were also considered as model small‐molecule drug cargos. However, data show inferior release dynamics compared to sodium fluorescein under constant irradiation for 2 h (λ = 630 nm) (Figure S7). We postulate that this is likely due to the higher molecular weight of these dyes (M_w_ = 691.85 and 479.01 g mol^−1^, for eosin Y disodium salt and rhodamine B, respectively, compared to 376.27 g mol^−1^ for sodium fluorescein). The molecular weights of the dyes are consistent with the observed release behaviors, with sodium fluorescein showing the highest relative dye release, followed by rhodamine B, while no clear difference was observed between irradiated samples and a dark control for the release of eosin Y. In addition to this, differences in the ionic nature of eosin Y and rhodamine B will influence the partitioning behavior of each dye in the polymersomes, meaning they are likely to localize within the lumen/membrane differently compared with sodium fluorescein. For this reason, sodium fluorescein was identified as a good model small molecule cargo, as it facilitated successful encapsulation and release from the polymersome carrier system.

As previously reported [41], upon excitation of the DASA polymer, the fluorescein cargo was released into the aqueous supernatant, diluting the concentration below the self‐quenching concentrations within the particles, and facilitating measurement of cargo release in the supernatant via an increase in fluorescence intensity from the fluorescein. We investigated two different modes of fluorescein release from aqueous dispersions of cargo‐laden polymersomes: samples were either kept in the dark or irradiated continuously at λ = 630 nm. When the dispersion was kept in the dark, the fluorescence intensity of fluorescein at λ = 518 nm remained unchanged, suggesting minimal release of the fluorescein cargo. In contrast, when the DASA polymersomes were irradiated, the fluorescence emission at λ = 518 nm increased during irradiation, indicative of cargo release from the DASA polymersomes (Figure 2d).

**FIGURE 3:**
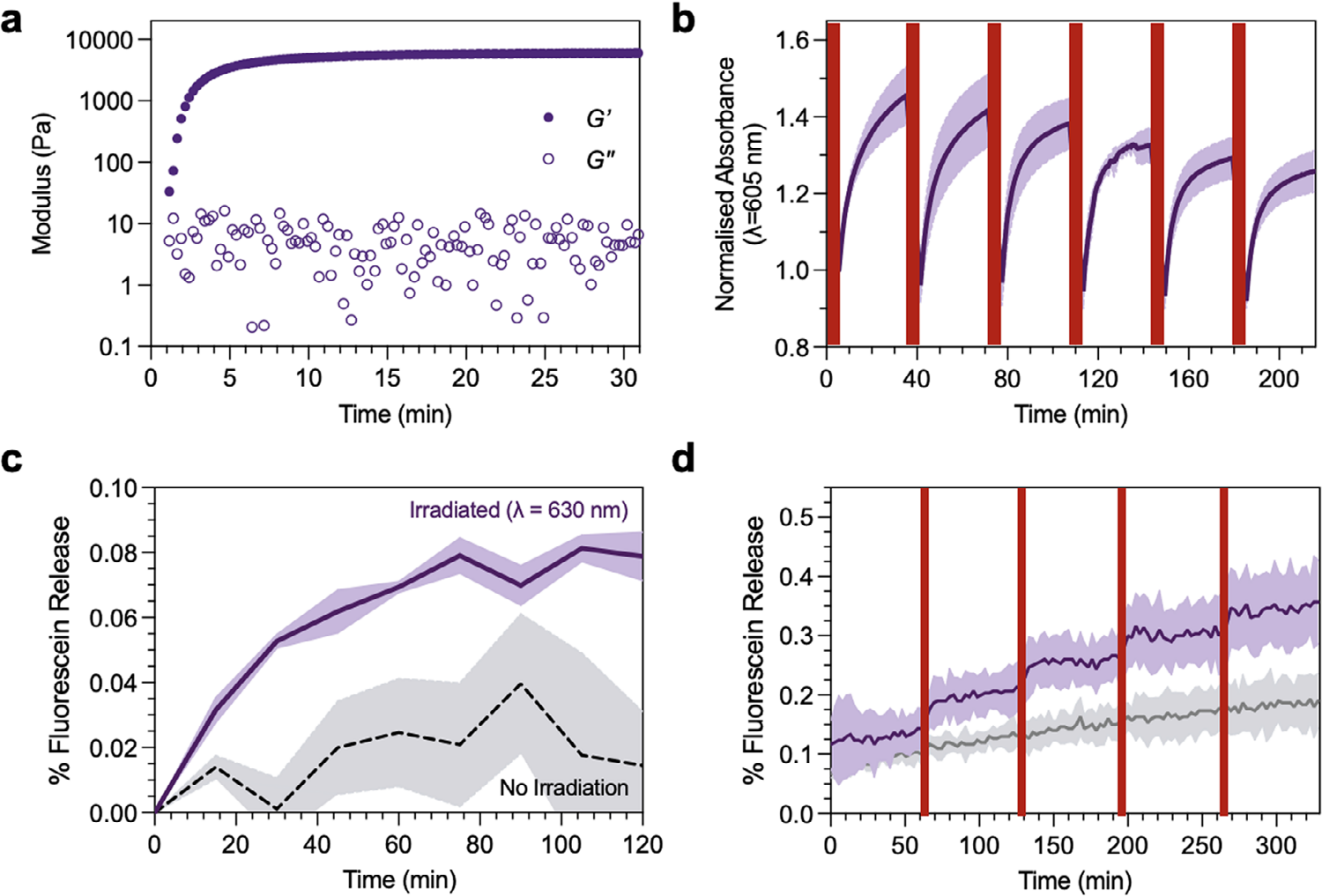
Photoswitching of DASA polymersomes encapsulated in a 4‐arm PEG DBCO‐azide hydrogel. (a) Time‐resolved rheology sweep, showing formation of the 10% w/v PEG‐DBCO‐azide gel upon polymerization of the resin. (b) Cyclic photoswitching and dark equilibration of the DASAs within the polymersome membranes when encapsulated in the polymer network. (n = 3, SD shown). (c) Fluorescence spectroscopy demonstrating light‐induced release of fluorescein from the hydrogel. Hydrogels were irradiated with visible light (λ = 630 nm), and cargo release was monitored at 15 min intervals over 120 min. This was compared to a control where the hydrogel was kept in the dark and baseline corrected (n = 3, SD shown). For quantification as concentration (nM) see Figure S8. (d) Cyclic switching experiment to investigate selective control of release rates. Hydrogels were fabricated by a click chemistry reaction between DBCO and azide‐functionalized PEG macromers in fluorescein‐loaded polymersome solution, then irradiated for 5 min with visible light (λ = 630 nm). Cargo release was then monitored by fluorescence spectroscopy at regular intervals for 1 h. This was repeated over 4 cycles and compared with a control where the hydrogels were kept in the dark (for Dye Release (%) calculations see Supporting Information). (n = 3, SD shown). This gave a maximum concentration of fluorescein loaded in the particles of 50.2 µM, corresponding to an encapsulation efficiency of 0.3%. For quantification as concentration (nM), see Figure S8.

Having demonstrated the suitability of the DASA polymersomes for the release of a small molecule cargo in aqueous conditions, we investigated whether the release of cargo could also be demonstrated in hydrogels. We used PEG hydrogels crosslinked via a strain‐promoted azide‐alkyne cycloaddition reaction between azide and dibenzocyclooctyne (DBCO) functional groups. We specifically used these hydrogels, as the method of crosslinking is orthogonal to any functional groups on the polymersomes (i.e., the triene‐enol of the DASA could act as a radical trap for radical polymerization‐derived hydrogels). To synthesize the hydrogels for polymersome retention, 4‐arm tetra‐amino poly(ethylene glycol) (PEG) macromers (M_w_ = 10 kDa) were functionalized with DBCO or azide functional groups via an amide coupling of a carboxylate derivative for strain‐promoted alkyne‐azide cycloadditions, characterized by ^1^H NMR and GPC (Figures S9–S11) [36]. This yielded 4‐arm PEG‐DBCO and PEG‐azide macromers that, upon mixing stoichiometrically, gave hydrogels with a highly controlled network structure, where the pore size is controlled through the molecular weight and branching structure of the PEG macromer (Figure 3a; Figure S12).

DASA polymersomes were retained in the PEG hydrogel matrix by dissolving the DBCO and azide macromers individually in polymersome dispersions, then mixing the two solutions (1:1, v/v) to crosslink the gels with the DASA polymersomes in situ. Time‐resolved rheology measurements showed a crossover point (storage modulus, *G′* > loss modulus, *G″*) after approximately 1 min, followed by a plateau in *G′* at 5.9 kPa after 5 min, which is three orders of magnitude greater than *G″*, indicating formation of a hydrogel (Figure 3a).

Photoswitching behavior was investigated via cyclic irradiation of the DASA polymersome‐laden hydrogel. Upon repeated cycles of irradiation (λ = 630 nm, 5 min) and equilibration (dark, 20°C, 30 min), recovery was demonstrated across six cycles as observed by measuring the absorbance at 605 nm of the triene‐enol form of the DASA after each cycle (86% recovery after six cycles, Figure 3b). This demonstrates that the photoswitching behavior of the DASA polymersomes was mostly conserved in the hydrogel matrix, with photoswitching characteristics similar to those of the DASA polymersomes in aqueous dispersion (Figure 2c). Fatigue between photoswitching cycles for the hydrogel‐encapsulated DASA polymersomes was expected due to interactions with the surrounding polymer matrix [45, 46]. The relaxation half‐life of the conjugated DASA was measured as *t_½_* = 10.8 min, suggesting longer dark cycles post‐irradiation could aid greater recovery (Figure S13). These studies also showed a range in the maximum absorbance of the DASA when encapsulated in the hydrogel, depending on the time post‐radiation. For photoswitching studies of the DASA polymersome‐laden hydrogels, we therefore used the maximum absorbance observed pre‐irradiation at λ = 605 nm.

After demonstrating reversible photo‐switching of the DASA‐polymersomes within the hydrogels, we sought to investigate whether we could demonstrate cargo release from the polymersomes. Fluorescence spectroscopy was used to track fluorescein release from the hydrogel into water. Dye release after 120 min was higher in irradiated samples. This demonstrated light‐triggered release of the fluorescein cargo from PEG DBCO‐azide hydrogels when subjected to continuous irradiation (Figure 3c).

Building upon this, further DASA‐hydrogels were fabricated in the same way. The ON‐OFF control of cargo release was investigated by cyclically irradiating samples with light (*t* = 15 min), to stimulate release of the fluorescent cargo, followed by a period in the dark (*t* = 60 min), to arrest release of the fluorescent cargo upon reversion to the “closed” cyclopentenone form of the DASA. When dye release was compared with the dark control, it showed rapid increases in release after irradiation, followed by equilibration of fluorescence in the supernatant during the dark intervals, suggesting no further release of the fluorescent dye. Upon each subsequent cycle of irradiation, fluorescence increased again, indicating that release of the dye from the polymersome‐laden hydrogel can be controlled by light (Figure 3d).

To our knowledge, this is the first report of a photoswitchable nanoparticle‐hydrogel system, showcasing a novel method for the targeted delivery of small‐molecule cargo by light‐triggered release. There is a notable difference in the percentage of fluorescein released between particles free in solution (0.7%) and those encapsulated within the hydrogel (0.4%). This is to be expected as the counts of fluorescence measured depend on the diffusion of fluorescein through the hydrogel network into the aqueous supernatant above, acting as a significant rate‐limiting step. Tuning of this rate of release will be essential for future work with this system, but suggests the ability to release over much longer timeframes. It is also important to consider the experimental setup for dye release experiments when discussing the differences observed between samples irradiated with 630 nm light and those treated as dark controls. As seen in Figure S6, these particles have high light sensitivity, with even ambient light exposure contributing to increases in cargo release. In this work, light exposure was minimized for the dark controls; transferring samples into the fluorimeter or changing samples between measurements would have caused ambient light exposure. Therefore, the total absence of light exposure was not possible for dark controls in Figure 3C. This may have contributed to the increase in fluorescence and larger error exhibited. Therefore, important considerations when repeating this work include establishing an experimental setup that allows for keeping samples in total darkness between measurements or irradiation. Preparing the particles immediately before measurement will also minimize issues with controlled release of cargo due to potential polymersome degradation over time.

Translation to delivery of small‐molecule therapeutic cargos would require careful optimization of pore size in the hydrogel to ensure efficient permeability of the cargo out of the hydrogel, as well as control of the interaction of the cargo with the polymersome membrane. We observed that different model cargos show different propensities for light‐triggered release from the polymersome system reported herein (Figure S7). As such, optimization of payload composition through careful tuning of molecular weight, ionic charge, and pH‐dependent effects would be important. These factors influence the partition coefficients of cargos, ultimately affecting their permeability by dictating whether they localize in the hydrophilic lumen or the hydrophobic membrane of the polymersomes. Further modification of small molecule therapeutic payloads to aid membrane permeability (promoting polymersome and cellular uptake) could be achieved, for example, through lipid‐conjugation as reported by Morstein et al. [47].

## 3 Conclusions and Future Perspectives

In this work, we show that polymersomes made from amphiphilic block copolymers with a light‐responsive DASA moiety in the hydrophobic block can be encapsulated into hydrogels for light‐controlled release of small molecule cargos. The polymersomes shielded the DASA from interactions with aqueous solvents through the self‐assembled supramolecular structure. Upon loading these polymersomes with a model small molecule cargo (fluorescein), visible light (λ = 630 nm) was shown to modulate the permeability of the membrane, facilitating cargo release. While 630 nm visible light does not achieve skin penetration depths of NIR wavelengths, it is already in routine clinical use in photodynamic therapy for oncology and wound management [48, 49, 50]. Furthermore, a recent breakthrough in improving the transmission of NIR light in rodent models through topical application of tartrazine dyes further widens the biological toolkit for use of light as an in vivo stimulant, and also provides notably higher transmission efficiency at 630 nm [51]. Building upon previous work, we introduced this light‐mediated release behavior into a hydrogel matrix via in situ encapsulation of the cargo‐laden nanoparticles. This was used to demonstrate ON‐OFF switchable cargo release using exposure of the system to repeated light‐dark cycles. With further optimization, this system could be applied to a wider range of small‐molecule cargos, including therapeutic cargos, setting a foundation for non‐invasive stimulation of materials for active, localized drug delivery.

We have demonstrated that loading photo‐responsive nanoparticles into hydrogels gives an elegant handle for controlled delivery of cargos. In the future, expanding the gamut of DASAs used to incorporate photoswitches that respond to different wavelengths of light could enable multi‐stimuli‐responsive, multiplexed systems with the potential to form more geometrically complex chemical gradients, based on orthogonal release of different cargos. This would hold future promise in diverse applications such as precise control of small‐scale chemical reactions and even recapitulation of the complex gradients of growth factors and nutrients seen in early tissue development and differentiation of stem cells.

## 4 Experimental

### 4.1 Materials

DBCO‐NHS ester was purchased from Lumiprobe (*Lumiprobe GmbH*, *Hannover*, *Germany*), and (poly)ethylene glycol (PEG) amine (PEG‐NH_2_) HCl salt was purchased from JenKem (*JenKem Technology USA Inc*., *TX*, *USA*). All other chemicals were purchased from Sigma–Aldrich (*Merck KGaA*, *Darmstadt*, *Germany)* and used without further purification. Unless otherwise stated, all solvents were purchased from VWR (*VWR International*, *LLC*., *PA*, *USA)* and used without further purification. UltraPure water was purchased from Invitrogen (*Invitrogen; Thermo Fisher Scientific*, *Inc*., *MA*, *USA*), and the MilliQ(R) Benchtop Lab Water Purification System was also used.

### 4.2 Synthetic methods

#### 4.2.1 Synthesis of DASA‐conjugated polymer: PEG‐*b*‐(PHMA‐*co*‐DASA)

##### 4.2.1.1 Synthesis of pentafluorophenyl methacrylate

This compound was synthesized following a procedure published by Théato and colleagues [52].

##### 4.2.1.2 Synthesis of poly(ethylene glycol)‐*b*‐(poly(hexyl acrylate)‐*co*‐poly(pentafluorophenyl methacrylate)) (PEG *b*‐(PHMA‐*co*‐PPFPMA)

Chain extension was carried out using reversible addition–fragmentation chain‐transfer (RAFT) radical polymerization on the macro chain transfer agent (macroCTA), poly(ethylene glycol)‐4‐cyano‐4‐(phenylcarbonothioylthio)pentanoate (PEG‐CPADB), to obtain an amphiphilic diblock copolymer incorporating randomly distributed activated ester groups. A mixture of PEG‐CPADB (121.8 mg, 48.7 µmol) and AIBN (1 mg, 6.09 µmol) was prepared in 0.77 mL of 1,4‐dioxane. In parallel, hexyl methacrylate (HMA) was purified to remove inhibitors by passing it through a basic alumina column to eliminate monomethyl ether hydroquinone. The initiator/macroCTA solution was then combined with pentafluorophenyl methacrylate (PFPMA; 44 µL, 61 mg, 0.24 mmol) (1) and the purified HMA (0.432 mL, 0.373 g, 2.19 mmol). The resulting reaction mixture was degassed under nitrogen for 1 h to eliminate dissolved oxygen. Polymerization was performed using a feed ratio of initiator:CTA:monomer = 1:8:400, with a monomer composition of HMA:PFPMA = 9:1 by molar ratio. The reaction proceeded at 90°C for 2 h, after which it was quenched by exposure to air. To purify the polymer, the reaction mixture was precipitated into a 60:40 methanol:water (v/v) solution. The precipitated material was collected by centrifugation at 5000 g for 15 min at room temperature. To further remove unreacted monomer, the pellet was redissolved in 2 mL of THF, and a small amount of Sudan Blue II was added as a molecular weight marker  for preparative size‐exclusion chromatography (SEC). SEC purification was carried out using Biobeads S‐X3 resin (fractionation range: 600–14 000 g·mol^−^^1^) with inhibitor‐free THF as the eluent. The polymer fraction was collected by monitoring elution prior to the appearance of the dye, then concentrated under reduced pressure. The purified product was subsequently dried in a vacuum oven at 40°C overnight, yielding 435 mg of purified polymer (78% yield).

##### 4.2.1.3 Removal of the chain‐transfer agent end group from PEG‐*b*‐(PHMA‐*co*‐PPFPMA)

The RAFT end group was cleaved from PEG‐*b*‐(PHMA‐*co*‐PPFPMA) following a modified thermal treatment protocol inspired by prior work [53]. Briefly, 360 mg of the copolymer was dissolved in 3 mL of DMF alongside AIBN (200 mg, 1.2 mmol) in a round‐bottom flask. The solution was degassed under a gentle nitrogen stream for 1 h to eliminate oxygen and then sealed under a nitrogen atmosphere to maintain inert conditions. Polymer end‐group removal was initiated by heating the reaction mixture in an 80°C oil bath for 4 h, allowing for thermal cleavage of the RAFT agent. After completion, the reaction mixture was cooled to room temperature. The crude polymer was purified by preparative size‐exclusion chromatography (SEC) using Biobeads S‐X3 resin (fractionation range: 600–14 000 g·mol^−^^1^) as the stationary phase and inhibitor‐free THF as the mobile phase. A small amount of Sudan Blue II was introduced as a molecular weight marker to identify the elution of small molecules. The purified polymer (3) was collected prior to the appearance of the dye fraction, then concentrated under reduced pressure. Finally, the polymer was dried overnight in a vacuum oven at 40°C to yield the RAFT‐cleaved copolymer.

##### 4.2.1.4 Synthesis of the second generation DASA donor precursor *N*‐(4‐methoxyphenyl)−1,3‐diaminopropane

The compound was synthesized following a procedure published by Han et al. [54].

##### 4.2.1.5 Synthesis of aromatic amine‐functionalized diblock copolymer

To introduce aromatic amine functionality, PEG‐*b*‐(PHMA‐*co*‐PPFPMA) capped with an AIBN‐derived end group was reacted with *N*‐(4‐methoxyphenyl)‐1,3‐diaminopropane. Specifically, the polymer was dissolved in 3 mL of anhydrous DMF alongside the amine reagent (100 mg, 0.55 mmol) and triethylamine (0.10 mL, 0.73 g, 1 mmol), which served to neutralize the resulting pentafluorophenol byproduct. The solution was degassed under a gentle nitrogen stream for 30 min to remove dissolved oxygen. The mixture was then sealed and incubated at 60°C under an inert atmosphere for 7 days, allowing sufficient time for nucleophilic substitution of the activated ester moieties by the aromatic diamine. Following the reaction, the product was purified via preparative SEC using Biobeads S‐X3 resin (fractionation range: 600–14 000 g·mol^−^^1^) and inhibitor‐free THF as the eluent. A trace amount of Sudan Blue II was used as a visible marker to differentiate small‐molecule fractions from polymeric material. Elution was monitored, and fractions collected prior to the appearance of the dye were pooled and concentrated under reduced pressure. The purified polymer (5) was then dried in a vacuum oven at 40°C overnight, yielding the functionalized diblock copolymer.

##### 4.2.1.6 Synthesis of 4‐(furan‐2‐ylmethylene)‐2,5‐diphenyl‐2,4‐dihydro‐3H‐pyrazol‐3‐one

The furan adduct was synthesized according to a method described by Rifaie–Graham et al. [41].

##### 4.2.1.7 Functionalization of the aromatic amine diblock copolymer with DASA moieties

The secondary aromatic amine groups on the functionalized diblock copolymer (5) were further reacted with a pyrazolone‐derived furan adduct to generate the corresponding DASA‐conjugated copolymer (7). In a typical reaction, 100 mg of the amine‐bearing polymer (approximately 12 µmol) was dissolved in a solution of the furan adduct (0.9 M) in 3 mL of a 1:9 HFIP:DCM solution in a round‐bottom flask. The flask was sealed with a rubber septum to maintain anhydrous conditions, and the mixture was stirred at room temperature for 7 days, allowing for the complete formation of the DASA chromophores via condensation. Upon completion of the reaction, excess furan adduct and small molecule byproducts were removed by preparative SEC using Biobeads S‐X3 resin (molecular weight range: 600–14 000 g·mol^−^^1^) and inhibitor‐free THF as the eluent. In this case, no tracking dye was required, as the distinct colors of the DASA‐modified polymer and unreacted furan adduct enabled visual monitoring of the separation. The purified product was then concentrated under reduced pressure and dried overnight at 40°C in a vacuum oven, affording the final DASA‐functionalized diblock copolymer.

#### 4.2.2 Synthesis of 4‐arm PEG‐DBCO

The precursor 4‐arm 10 kDa PEG‐NH_2_ HCl salt was used to synthesize 4‐arm 10 kDa PEG‐DBCO and PEG‐N_3_ through amide coupling reactions. To synthesize PEG‐DBCO, PEG‐NH_2_ (1000.23 mg), DBCO‐NHS ester (344.54 mg, 8 mol equiv.) was dissolved in *N*,*N*‐dimethylformamide (8 mL) and added to PEG‐NH_2_. Then, *N*,*N*‐diisopropylethyl amine (279 µL) was added to the reaction mixture, which was covered in aluminium foil and stirred at room temperature overnight. The reaction mixture was transferred to a Falcon tube^TM^ and made up to 50 mL with diethyl ether, then centrifuged at 5000 rcf for 5 min. The supernatant was removed, and the precipitate was left to dry under a gentle stream of N_2_. The solid was dissolved in ultrapure water (50 mL), and dialyzed using 6–8 kDa membranes, with the ultrapure water being changed five times over the course of two days. The solution was lyophilized and stored at −20°C.

In agreement with the literature [38], this synthesis was confirmed by ^1^H NMR (500 MHz, D_2_O, δ): 7.68‐7.34 (m, 21H, H^Ar^), 5.09 (d, *J* = 13.3 Hz, 2H, H^g^), 3.78‐3.59 (m, 997H, H^b^), 3.52‐3.47 (s, 8H, H^a^), 3.34‐3.29 (m, 5H, H^b’^), 2.20‐1.97 (m, 7H, H^c,f^), 1.28‐1.18 (m, 10H, H^d,e^) ppm. Isolated mass: >950 mg.

#### 4.2.3 Synthesis of 4‐arm PEG‐N_3_

The procedure was similar for the synthesis of PEG‐N_3_, but with azidoacetic acid (59.9 µL), NHS (92.14 mg), and DIC (119 µL) being added to the reaction mixture instead of DBCO‐NHS.

This synthesis was also confirmed by ^1^H NMR (500 MHz, D_2_O, δ): 4.04 (s, 7H, H^d^), 3.83‐3.60 (m, 1067H, H^b^), 3.51 (s, 8H, H^a^), 3.48‐3.45 (t, *J* = 5.4 Hz, 7H, H^c^) ppm. Isolated mass: >690 mg.

### 4.3 Self‐assembly of DASA‐polymersomes by solvent exchange

PEG‐*b*‐(PHMA‐*co*‐DASA) (2.5 mg) was dissolved in THF (1.25 mL) in a 7 mL glass vial and left stirring with a magnetic stirrer bar for 10 min. Once dissolved, the solution (1 mL) was divided into two new 7 mL glass vials, each with a magnetic stirrer bar to continue stirring. A solution of fluorescein sodium salt (43.7 mg) in ultrapure deionized water (5 mL) was added via syringe to one vial using a syringe pump at a flow rate of 400 µL min^−1^. The mixture was left stirring for 10 min, then transferred to a Pur‐A‐Lyzer 1 kDa Mega 1000 dialysis tube and left for seven days, changing the water once daily.

### 4.4 Hydrogel preparation

A total of 10% w/v 4‐arm PEG functionalized with DBCO (PEG‐DBCO) and with azide (PEG‐azide) was dissolved in a dispersion of DASA polymersomes, either empty or loaded with fluorescein. The two PEG solutions were mixed to trigger crosslinking of the macromers (complete within 5 min) to form hydrogels loaded with either empty or fluorescein‐loaded DASA particles, respectively.

### 4.5 Analytical techniques

#### 4.5.1 Dynamic light scattering (DLS) on DASA‐polymersomes

Samples (500 µL) were measured using a Malvern Zetasizer Nano‐ZS (*Malvern Panalytical*, *UK*) in ZEN0040 disposable cuvettes (1.5 mL). Size measurements were taken in triplicate (*n* = 3) by NIBS at a 173° scattering angle with a 30 s equilibration time at 25°C.

#### 4.5.2 UV–Vis on DASA‐polymersomes

Samples were measured using a UV–vis NanoDrop 2000c Spectrophotometer (*Thermo Scientific*, *Inc*., *MA*, *USA*). Each sample (500 µL) was added to a ZEN0040 disposable cuvette (1.5 mL) and measured in cuvette mode (1 cm path length) against an ultrapure water blank.

#### 4.5.3 Nuclear magnetic resonance (NMR)

##### 4.5.3.1 NMR of the DASA‐conjugated polymer

NMR spectra were recorded at 298 K using a Bruker AvanceNEO 600 spectrometer (*Bruker*, *UK Ltd*., *Coventry*, *UK*). Residual solvent peaks were used as an internal reference (CDCl_3_).

##### 4.5.3.2 NMR of PEG‐azide and PEG‐DBCO

NMR samples (10–20 mg mL^−1^) were made up in D_2_O for characterization by ^1^H NMR using a 500 MHz Bruker Avance II NMR spectrometer (*Bruker*, *UK Ltd*., *Coventry*, *UK*), a room temperature broadband probe, Oxford Instruments Company magnet (*Oxford Instruments plc*, *Oxfordshire*, *UK*) and TopSpin software (*version 3.1.7, Bruker*, *UK Ltd*., *Coventry*, *UK*). Deuterated trimethylsilyl propionic acid*‐d_4_ (*TMSP‐*d_4_)* was used as an internal standard. Spectra were collected at 298 K, using 16 scans and a 10 s relaxation delay. Spectra were corrected to the chemical shift of the solvent peak (D_2_O: 4.79 ppm), and further data analysis was performed on MestReNova (version 15.0.1‐35756, 2024, *Mestrelab Research S.L.U*., *Santiago de Compostela*, *Spain*).

#### 4.5.4 Hydrogel characterization by rheology

Time‐resolved rheology was carried out on hydrogels that were polymerized in situ on an Anton Paar modular compact rheometer (MCR302) with a PP08 stainless‐steel parallel plate (both *Anton Paar GmbH*, *Graz*, *Austria*). A constant frequency = 1.00 Hz and a strain = 0.1% were used in the time‐resolved rheology measurements. This strain was chosen as it was within the linear viscoelastic region of the hydrogel, whereas the hydrogel showed 𝐺′ ≫ 𝐺′′ at a frequency of 1 Hz. Additionally, a frequency sweep under constant strain (0.1%) and a strain sweep under constant frequency (1 Hz) were measured to understand the long‐term stability and structural strength of the material, respectively (Figure S13).

#### 4.5.5 Gel permeation chromatography (GPC)

##### 4.5.5.1 PEG‐*b*‐(PHMA‐*co*‐DASA) GPC

The molecular weight distributions were determined by GPC using the Agilent 1260 Infinity Multi‐Detector Suite instrument (*Agilent Technologies LDA UK Ltd*., *Stockport, UK*), equipped with two PLgel MIXED ‐C 5 µm columns, refractive index, and UV detectors. The mobile phase used in the instrument is GPC‐grade tetrahydrofuran (THF) at a flow rate of 1 mL min^−1^ (Figure S1).

##### 4.5.5.2 PEG‐azide and PEG‐DBCO GPC

GPC samples were made up at a concentration of 5–10 mg mL^−1^ in DMF spiked with 0.075% w/w LiBr (1 mL min^−1^, flow rate, 40°C) and filtered through 0.2 µm PTFE filters, then added to Supelco vials for measurement (Figure S12). An internal calibration was performed prior to measurements using 12 poly(methyl methacrylate) standards: 535, 1015, 1820, 6960, 13 630, 33 870, 60 300, 156 200, 273 600, 536 500, 1226 000, and 1795 000 g mol^‒1^ (PMMA EasiVials from *Agilent Technologies LDA UK Ltd*., *Stockport, UK*). The GPC has two in‐line GRAM linear columns (10 µm, 8 × 300 mm) and one GRAM guard column (10 µm, 8 × 50 mm^2^).

#### 4.5.6 Irradiation of DASA polymersomes

Light irradiation of the DASA polymersomes and DASA polymersomes in hydrogels was carried out using an LED array with an excitation wavelength λ = 630 nm (LEDA‐X LED array; *Teleopto*, *Bio Research Center Co*., *Ltd*., *Nagoya*, *Japan*) connected to an LED array driver unit (LAD‐1 LED array driver; *Teleopto*, *Bio Research Center Co*., *Ltd*., *Nagoya*, *Japan*).

#### 4.5.7 Characterization of photoswitching in DASA polymersomes

##### 4.5.7.1 Cyclic switching in aqueous dispersion

Dispersions of PEG‐*b*‐(PHMA‐*co*‐DASA) polymersomes were prepared at 0.1, 0.2, and 0.4 mg mL^−1^ in PBS (n = 3). Each dispersion was irradiated for 5 min at 5.4 mW cm^−2^ (λ = 630 nm). To allow for switching from the “closed” cyclopentenone form to its “open” triene‐enol form, samples were left in the absence of light for 30 min. Recovery was monitored by tracking the absorbance at λ = 630 nm of the “open” triene‐enol DASA at 630 nm using a PerkinElmer EnVision multimode microplate reader (*PerkinElmer*, *CT*, *USA*). This process was repeated over four cycles.

##### 4.5.7.2 Cyclic switching in hydrogels

PEG‐DBCO and PEG‐azide were dissolved in empty polymersome solution (100 mg mL^−1^), mixed in equal stoichiometries (1:1, v/v), then allowed to cure in a 96‐well plate to form 100 µL gels (*N* = 3). Each gel was crosslinked for 5 min, then immersed in ultrapure deionized water (100 µL). Cyclic switching studies were carried out by measuring the absorbance (λ = 605 nm) of the DASA tautomer using a Spectramax M5 UV–vis spectrometer (*Molecular Devices*, *LLC*., *CA*, *USA)*. Each gel was irradiated for 5 min at 5.4 mW cm^−2^ (λ = 630 nm). It was then allowed to revert from its “closed” cyclopentenone form to its “open” triene‐enol form over 30 min in the dark. Measurements were acquired every 30 s during this period and repeated over six cycles.

#### 4.5.8 Measurement of fluorescent dye release by fluorescence spectroscopy

##### 4.5.8.1 Fluorescein release in aqueous dispersion

Fluorescein disodium salt was encapsulated in polymersomes by solvent exchange. Aqueous dispersions were dialyzed for 3 days into deionized water to remove any non‐encapsulated dye using a pre‐wetted Spectra/Por 6 reconstituted cellulose dialysis membrane (MWCO = 1 kDa) (*Repligen Corp*., *MA*, *USA*). Polymersomes were diluted 30 times in UltraPure water and transferred to a Fisherbrand 1.5 mL PMMA disposable fluorescence cuvette (*Thermo Fisher Scientific*, *Inc*., *MA*, *USA*). Samples were irradiated continuously (λ = 630 nm) and fluorescence was measured at regular intervals (λ_ex_ = 493 nm, λ_em_ = 518 nm). Control samples were prepared in the same manner and kept in the dark, with their fluorescence monitored across the same period.

##### 4.5.8.2 Eosin Y and Rhodamine B release in aqueous dispersion

Eosin Y disodium salt and rhodamine B were encapsulated in polymersomes by solvent exchange, using the protocol described above, with 25 mM of the dye in the aqueous phase. Aqueous dispersions were dialyzed for 7 days into deionized water to remove any non‐encapsulated dye using a pre‐wetted Spectra/Por 1 reconstituted cellulose dialysis membrane (MWCO = 6–8 kDa) (*Repligen Corp*., *MA*, *USA*). Polymersomes were diluted 30 times in UltraPure water and transferred to a Fisherbrand 1.5 mL PMMA disposable fluorescence cuvette (*Thermo Fisher Scientific*, *Inc*., *MA*, *USA*). Samples were irradiated continuously (λ = 630 nm) and fluorescence was measured at regular intervals (λ_ex_ = 525 nm, λ_em_ = 545 nm for Eosin Y and λ_ex_ = 546 nm, λ_em_ = 567 nm for rhodamine B). Control samples were prepared in the same manner and kept in the dark, with their fluorescence monitored across the same period.

##### 4.5.8.3 Continuous fluorescein release from polymersome‐encapsulating hydrogels

DASA‐polymersome encapsulating hydrogels were transferred to a quartz cuvette or Fisherbrand 1.5 mL PMMA disposable fluorescence cuvette (*Thermo Fisher Scientific*, *Inc*., *MA*, *USA*) and immersed in a four times volumetric excess of ultrapure deionized water. Samples were irradiated continuously at 630 nm, taking measurements at regular 15 min intervals (*N* = 3). Fluorescence (λ_ex_ = 493 nm, λ_em_ = 518 nm) was measured using a Horiba Fluorolog fluorometer (*Horiba Ltd*., *Kyoto*, *Japan*). Control samples were kept in the dark, with fluorescence monitored over the same period (*N* = 3).

##### 4.5.8.4 Cyclic fluorescein release from polymersome‐encapsulating hydrogels

DASA‐polymersome encapsulating hydrogels were transferred to a quartz cuvette or Fisherbrand 1.5 mL PMMA disposable fluorescence cuvette (*Thermo Fisher Scientific*, *Inc*., *MA*, *USA*) and immersed in a five times volumetric excess of ultrapure deionized water. Samples were irradiated for 5 min (λ = 630 nm), with fluorescence subsequently monitored at regular intervals for 1 h. This was repeated across four cycles. Control samples were kept in the absence of light, with fluorescence monitored over the same period. The fluorescence of the supernatant was measured using a Horiba Fluorolog fluorometer (*Horiba Ltd*., *Kyoto*, *Japan*) (λ_ex_ = 493 nm, λ_em_ = 518 nm). Change (Δ) in fluorescence release was calculated by subtracting the dark control from the irradiated samples to isolate the effects of light exposure (λ = 630 nm) on particle release.

#### 4.5.9 Cryo‐TEM of DASA polymersomes

DASA‐polymersomes were prepared via solvent exchange using 4.0 mg (PEG‐*b*‐(PHMA‐*co*‐DASA)) dissolved in 200 µL THF, and 2 mL UltraPure water was added whilst stirring, then left to stir for 10 min. An aliquot of sample (4 µL) was adsorbed onto a glow‐discharged (*Qorum*, *UK*) holey carbon‐coated grid (*Lacey*, *Tedpella*, *USA*) for 60 s, blotted 4 s with Whatman grade 1 filter paper, and vitrified into liquid ethane at −180°C using a Leica GP2 plunger (*Leica microsystems*, *Austria*). Frozen grids were transferred onto a Talos 200C Electron microscope (*FEI*, *USA*) operating at 200 KV. The micrographs were acquired with a CETA CMOS detector using low dose system (electron dose = 40 e^−^ Å^−2^) at a magnification of 74 000×, corresponding to 2.2 Å per pixel on the image (corresponding to the scale bar). Defocus values were −2 to −3 µm.

## Author Contributions

Conceptualization: Tristan N. Dell, Ana Cammack‐Najera, Omar Rifaie‐Graham, Jonathan P. Wojciechowski, Molly M. Stevens. Methodology: Tristan N. Dell, Ana Cammack‐Najera, Ray G. DiNardi, Omar Rifaie‐Graham, Jonathan P. Wojciechowski, Molly M. Stevens. Investigation: Tristan N. Dell, Ana Cammack‐Najera, Omar Rifaie‐Graham, Jonathan P. Wojciechowski, Molly M. Stevens. Synthesis: Rea Tresa, Farzina Matubbar, Beyzanur Kaya, Omar Rifaie‐Graham. Characterization: Tristan N. Dell, Ana Cammack‐Najera, Rea Tresa, Farzina Matubbar, Beyzanur Kaya, Uthaya Lathan, Mohamed Chami, Omar Rifaie‐Graham, Jonathan P. Wojciechowski. Supervision: Omar Rifaie‐Graham, Jonathan P. Wojciechowski, Molly M. Stevens. Writing – original draft: Tristan N. Dell, Ana Cammack‐Najera, Jonathan P. Wojciechowski. Writing – review and editing: Tristan N. Dell, Ana Cammack‐Najera, Rea Tresa, Farzina Matubbar, Beyzanur Kaya, Uthaya Lathan, Mohamed Chami, Ray G. DiNardi, Omar Rifaie‐Graham, Jonathan P. Wojciechowski, Molly M. Stevens.

## Conflicts of Interest

M.M.S. has invested in, consults for (or is on scientific advisory boards or boards of directors), and conducts sponsored research funded by companies related to the biomaterials field; has filed patent applications related to biomaterials; and has co‐founded companies in the biomaterials field. The rest of the authors declare no conflict of interest.

## Supporting information




**Supporting File**: marc70218‐sup‐0001‐SuppMat.docx.

## Data Availability

The data that support the findings of this study are available from the corresponding author upon reasonable request.

## References

[1] M. H. Cai , et al., “Design and Development of Hybrid Hydrogels for Biomedical Applications: Recent Trends in Anticancer Drug Delivery and Tissue Engineering,” Front Bioengineering Biotechnology 9 (2021): 630943. 10.3389/fbioe.2021.630943 PMC7925894 33681168

[2] Y. Cao , R. Xie , P. W. A. Schönhöfer , et al., “Permanent Magnetic Droplet–Derived Microrobots,” Science Advances 11 (2025): adw3172, 10.1126/sciadv.adw3172. PMC12239960 40632849

[3] Z. Xia , B. Guo , D. Wu , F. Yang , and Y. Ding , “Advances of Natural Hydrogel‐based Vascularization Strategies for Soft Tissue Repair,” Frontiers in Materials 11 (2024): 1446035, 10.3389/fmats.2024.1446035.

[4] J. Li and D. J. Mooney , “Designing Hydrogels for Controlled Drug Delivery,” Nature Reviews Materials 1 (2016): 16071, 10.1038/natrevmats.2016.71. PMC5898614 29657852

[5] S. R. Caliari and J. A. Burdick , “A Practical Guide to Hydrogels for Cell Culture,” Nature Methods 13 (2016): 405–414, 10.1038/nmeth.3839. 27123816 PMC5800304

[6] T. H. Qazi , M. R. Blatchley , M. D. Davidson , et al., “Programming Hydrogels to Probe Spatiotemporal Cell Biology,” Cell Stem Cell 29 (2022): 678–691, 10.1016/j.stem.2022.03.013. 35413278 PMC9081204

[7] S. Tang , B. M. Richardson , and K. S. Anseth , “Dynamic Covalent Hydrogels as Biomaterials to Mimic the Viscoelasticity of Soft Tissues,” Progress in Materials Science 120 (2021): 100738, 10.1016/j.pmatsci.2020.100738.

[8] R. Tomer and A. T. Florence , “Photo‐Responsive Hydrogels For Potential Responsive Release Applications,” International Journal of Pharmaceutics 99 (1993): R5–R8, 10.1016/0378-5173(93)90383-Q.

[9] S. Matsumoto , S. Yamaguchi , S. Ueno , et al., “Photo Gel–Sol/Sol–Gel Transition and Its Patterning of a Supramolecular Hydrogel as Stimuli‐Responsive Biomaterials,” Chemistry—A European Journal 14 (2008): 3977–3986, 10.1002/chem.200701904. 18335444

[10] D. R. Griffin , J. T. Patterson , and A. M. Kasko , “Photodegradation as a Mechanism for Controlled Drug Delivery,” Biotechnology and Bioengineering 107 (2010): 1012–1019, 10.1002/bit.22882. 20661910

[11] P. Xing and Y. Zhao , “Supramolecular Vesicles for Stimulus‐Responsive Drug Delivery,” Small Methods 2 (2018): 1700364, 10.1002/smtd.201700364.

[12] S. Mura , J. Nicolas , and P. Couvreur , “Stimuli‐Responsive Nanocarriers for Drug Delivery,” Nature Materials 12 (2013): 991–1003, 10.1038/nmat3776. 24150417

[13] C. Thanapongpibul , O. Rifaie‐Graham , M. Ojansivu , et al., “Unlocking Intracellular Protein Delivery by Harnessing Polymersomes Synthesized at Microliter Volumes using Photo‐PISA,” Advanced Materials 36 (2024): 2408000, 10.1002/adma.202408000. 39417762 PMC11619233

[14] M. R. Molla , P. Rangadurai , L. Antony , S. Swaminathan , J. J. de Pablo , and S. Thayumanavan , “Dynamic Actuation of Glassy Polymersomes Through Isomerization of a Single Azobenzene Unit at the Block Copolymer Interface,” Nature Chemistry 10 (2018): 659–666, 10.1038/s41557-018-0027-6. 29713034

[15] D. Liu , S. Wang , S. Xu , and H. Liu , “Photocontrollable Intermittent Release of Doxorubicin Hydrochloride From Liposomes Embedded by Azobenzene‐Contained Glycolipid,” Langmuir 33 (2017): 1004–1012, 10.1021/acs.langmuir.6b03051. 27668306

[16] K. Lützel , et al., “A Platform for the Development of Highly Red‐Shifted Azobenzene‐Based Optical Tools,” Angewandte Chemie International Edition 64 (2025): 202501779. 10.1002/anie.202501779 PMC12322654 40464072

[17] S. Menon , A. Krishnan , and S. Roy , “Engineering Azo Compounds for Visible and NIR Light Responsiveness: Taking a Molecular Point of View,” Materials Today Chemistry 47 (2025): 102836, 10.1016/j.mtchem.2025.102836.

[18] F. A. Jerca , V. V. Jerca , and R. Hoogenboom , “Advances and Opportunities in the Exciting World of Azobenzenes,” Nature Reviews Chemistry 6 (2022): 51–69, 10.1038/s41570-021-00334-w. 37117615

[19] C. Yao , P. Wang , X. Li , et al., “Near‐Infrared‐Triggered Azobenzene‐Liposome/Upconversion Nanoparticle Hybrid Vesicles for Remotely Controlled Drug Delivery to Overcome Cancer Multidrug Resistance,” Advanced Materials 28 (2016): 9341–9348, 10.1002/adma.201503799. 27578301

[20] J. Liu , W. Bu , L. Pan , and J. Shi , “NIR‐Triggered Anticancer Drug Delivery by Upconverting Nanoparticles With Integrated Azobenzene‐Modified Mesoporous Silica,” Angewandte Chemie 125 (2013): 4471–4475, 10.1002/ange.201300183. 23495013

[21] Y. Niu , Y. Li , Y. Lu , and W. Xu , “Spiropyran‐Decorated Light‐Responsive Amphiphilic Poly(α‐hydroxy acids) Micelles Constructed via a CuAAC Reaction,” RSC Advances 4 (2014): 58432–58439, 10.1039/C4RA11550C.

[22] S. Son , E. Shin , and B. S. Kim , “Light‐Responsive Micelles Of Spiropyran Initiated Hyperbranched Polyglycerol For Smart Drug Delivery,” Biomacromolecules 15 (2014): 628–634, 10.1021/bm401670t. 24432713

[23] X. Wang , J. Hu , G. Liu , et al., “Reversibly Switching Bilayer Permeability and Release Modules of Photochromic Polymersomes Stabilized by Cooperative Noncovalent Interactions,” Journal of the American Chemical Society 137 (2015): 15262–15275, 10.1021/jacs.5b10127. 26583385

[24] R. Klajn , “Spiropyran‐Based Dynamic Materials,” Chemical Society Reviews 43 (2014): 148–184, 10.1039/C3CS60181A. 23979515

[25] A. Fagan , M. Bartkowski , and S. Giordani , “Spiropyran‐Based Drug Delivery Systems,” Frontiers in Chemistry 9 (2021): 720087, 10.3389/fchem.2021.720087. 34395385 PMC8358077

[26] R. Sun , X. Song , K. Zhou , et al., “Assembly of Fillable Microrobotic Systems by Microfluidic Loading With Dip Sealing,” Advanced Materials 35 (2023): 2207791, 10.1002/adma.202207791. PMC7615483 36502366

[27] T. Lin , J. Yang , Y. Li , et al., “Near‐Infrared‐Driven Metal–Organic Frameworks‐Based Nanorobots for Controlled Photothermal‐Chemical Therapy Synergistic Induction of Cancer Cell Death,” Advanced Robotics Research (2025): 202500104, 10.1002/adrr.202500104.

[28] C. Sheng , F. Yu , Y. Feng , et al., “Near‐Infrared Light Triggered Degradation Of Metal−Organic Frameworks For Spatiotemporally‐Controlled Protein Release,” Nano Today 49 (2023): 101821, 10.1016/j.nantod.2023.101821.

[29] M. Sharifi , F. Attar , A. A. Saboury , et al., “Plasmonic Gold Nanoparticles: Optical Manipulation, Imaging, Drug Delivery and Therapy,” Journal of Controlled Release 311 (2019): 170–189, 10.1016/j.jconrel.2019.08.032. 31472191

[30] Y. Han , X. Yang , Y. Liu , et al., “Supramolecular Controlled Cargo Release via Near Infrared Tunable Cucurbit[7]Uril‐Gold Nanostars,” Scientific Reports 6 (2016): 22239, 10.1038/srep22239. 26917240 PMC4768098

[31] M. Clerc , S. Sandlass , O. Rifaie‐Graham , et al., “Visible Light‐Responsive Materials: The (photo)Chemistry And Applications Of Donor–Acceptor Stenhouse Adducts In Polymer Science,” Chemical Society Reviews 52 (2023): 8245–8294, 10.1039/D3CS00508A. 37905554 PMC10680135

[32] S. Helmy , F. A. Leibfarth , S. Oh , J. E. Poelma , C. J. Hawker , and J. Read de Alaniz , “Photoswitching Using Visible Light: A New Class of Organic Photochromic Molecules,” Journal of the American Chemical Society 136 (2014): 8169–8172, 10.1021/ja503016b. 24848124

[33] J. R. Hemmer , S. O. Poelma , N. Treat , et al., “Tunable Visible and Near Infrared Photoswitches,” Journal of the American Chemical Society 138 (2016): 13960–13966, 10.1021/jacs.6b07434. 27700083

[34] J. R. Hemmer , Z. A. Page , K. D. Clark , et al., “Controlling Dark Equilibria and Enhancing Donor–Acceptor Stenhouse Adduct Photoswitching Properties Through Carbon Acid Design,” Journal of the American Chemical Society 140 (2018): 10425–10429, 10.1021/jacs.8b06067. 30074782

[35] F. Stricker , D. M. Sanchez , U. Raucci , et al., “A Multi‐stage Single Photochrome System for Controlled Photoswitching Responses,” Nature Chemistry 14 (2022): 942–948, 10.1038/s41557-022-00947-8. 35681046

[36] C. Hu , Y. Sun , G. van Wissen , Y. Peng , and A. Pich , “Visible Light‐Responsive Microgels Modified With Donor–Acceptor Stenhouse Adducts,” Chemistry of Materials 34 (2022): 4774–4784, 10.1021/acs.chemmater.2c00969.

[37] Y. Ling , Y. Dong , W. Huang , J. Liu , S. Feng , and W. Huang , “Orthogonally Responsive Donor–Acceptor Stenhouse Adduct/Poly(2‐ethylsulfonyl‐2‐oxazoline) Colorimetric Sensors With Nonvolatile Memories,” ACS Applied Polymer Materials 4 (2022): 6505–6513, 10.1021/acsapm.2c00915.

[38] Y. Dong , Y. Ling , D. Wang , et al., “Harnessing Molecular Isomerization in Polymer Gels for Sequential Logic Encryption and Anticounterfeiting,” Science Advances 8 (2022): add1980, 10.1126/sciadv.add1980. PMC9629717 36322650

[39] S. Ulrich , et al., “Nano‐3D‐Printed Photochromic Micro‐Objects,” Small 17 (2021): 2101337. 10.1002/smll.202101337 34028975

[40] L. F. Muff , et al., “Pendent No More: Direct Backbone Integration of Stenhouse Salt Enables Multi‐Responsive Commodity Polyurethanes,” Angewandte Chemie International Edition 64 (2025): 202514632. 10.1002/anie.202514632 41123268

[41] O. Rifaie‐Graham , S. Ulrich , N. F. B. Galensowske , et al., “Wavelength‐Selective Light‐Responsive DASA‐Functionalized Polymersome Nanoreactors,” Journal of the American Chemical Society 140 (2018): 8027–8036, 10.1021/jacs.8b04511. 29856216

[42] O. Rifaie‐Graham , J. Yeow , A. Najer , et al., “Photoswitchable Gating of Non‐equilibrium Enzymatic Feedback in Chemically Communicating Polymersome Nanoreactors,” Nature Chemistry 15 (2023): 110–118, 10.1038/s41557-022-01062-4. PMC9836937 36344820

[43] F. López Arbeloa , P. R. Ojeda , and I. López Arbeloa , “flourescence Self‐Quenching Of The Molecular Forms Of Rhodamine B In Aqueous And Ethanolic Solutions,” Journal of Luminescence 44 (1989): 105–112, 10.1016/0022-2313(89)90027-6.

[44] C. Munkholm , D.‐R. Parkinson , and D. R. Walt , “Intramolecular Fluorescence Self‐Quenching of Fluoresceinamine,” Archives of Biochemistry and Biophysics 112 (1990): 66–69.

[45] R. McDonough , et al., “Fatigue of Donor‐Acceptor Stenhouse Adducts in Polymer Matrices and Solution,” ChemPhotoChem 7 (2023): 202200247.

[46] J. P. Wesseler , G. M. Cameron , P. A. G. Cormack , and N. Bruns , “donor–Acceptor Stenhouse Adduct Functionalised Polymer Microspheres,” Polymer Chemistry 14 (2023): 1456–1468, 10.1039/D2PY01591A. 37009639 PMC10043757

[47] J. Morstein , A. Capecchi , K. Hinnah , et al., “Medium‐Chain Lipid Conjugation Facilitates Cell‐Permeability and Bioactivity,” Journal of the American Chemical Society 144 (2022): 18532–18544, 10.1021/jacs.2c07833. 36178375

[48] N. Sultana , R. Pathak , S. Samanta , and N. Sen Sarma , “A Comprehensive Analysis of Photothermal Therapy (PTT) and Photodynamic Therapy (PDT) for the Treatment of Cancer,” Process Biochemistry 148 (2025): 17–31, 10.1016/j.procbio.2024.11.015.

[49] X. Li , J. F. Lovell , J. Yoon , and X. Chen , “Clinical Development and Potential of Photothermal and Photodynamic Therapies for Cancer,” Nature Reviews Clinical Oncology 17 (2020): 657–674, 10.1038/s41571-020-0410-2. 32699309

[50] R. Mancusi , G. Nosso , S. Pecoraro , M. Barricelli , and A. Russo , “Photodynamic Therapy with RLP068 and 630‐nm Red LED Light in Foot Ulcers in Patients with Diabetes: A Case Series,” The International Journal of Lower Extremity Wounds 23 (2024): 99–103, 10.1177/15347346211053403. 34693762

[51] Z. Ou , Y.‐S. Duh , N. J. Rommelfanger , et al., “Achieving Optical Transparency in Live Animals With Absorbing Molecules,” Science 385 (2024): 1061, 10.1126/science.adm6869. PMC11931656 39236186

[52] M. Eberhardt , R. Mruk , R. Zentel , and P. Théato , “Synthesis of Pentafluorophenyl(meth)Acrylate Polymers: New Precursor Polymers for the Synthesis of Multifunctional Materials,” European Polymer Journal 41 (2005): 1569–1575, 10.1016/j.eurpolymj.2005.01.025.

[53] S. Perrier , P. Takolpuckdee , and C. A. Mars , “Reversible Addition−Fragmentation Chain Transfer Polymerization: End Group Modification for Functionalized Polymers and Chain Transfer Agent Recovery,” Macromolecules 38 (2005): 2033–2036, 10.1021/ma047611m.

[54] H. Yin , M. Jin , W. Chen , et al., “Solvent‐Free Copper‐catalyzed N‐arylation of Amino Alcohols and Diamines With Aryl Halides,” Tetrahedron Letters 53 (2012): 1265–1270, 10.1016/j.tetlet.2011.12.130.

