# Supplementary material for: Hydrogels Incorporating Donor–Acceptor Stenhouse Adducts as a Platform for Photoinduced, On‐Off Switchable Release of Small Molecule Cargos
Source: Macromol Rapid Commun. 2026 Jan 31;47(8):e00868. doi: 10.1002/marc.202500868 (PMC13087835; doi:10.1002/marc.202500868)
Supplement: Supplementary file 1 — Supporting File: marc70218‐sup‐0001‐SuppMat.docx. [file MARC-47-e00868-s001.docx]

Supporting Information

Hydrogels Incorporating Donor-Acceptor Stenhouse Adducts as a Platform for Photoinduced, On-Off Switchable Release of Small Molecule Cargos

*Tristan N. Dell,^†^ Ana Cammack-Najera,^†^ Rea Tresa, Farzina Matubbar, Beyzanur Kaya, Uthaya Lathan, Mohamed Chami, Ray G. DiNardi, Omar Rifaie-Graham,* Jonathan P. Wojciechowski,* Molly M. Stevens**

**General Notes**


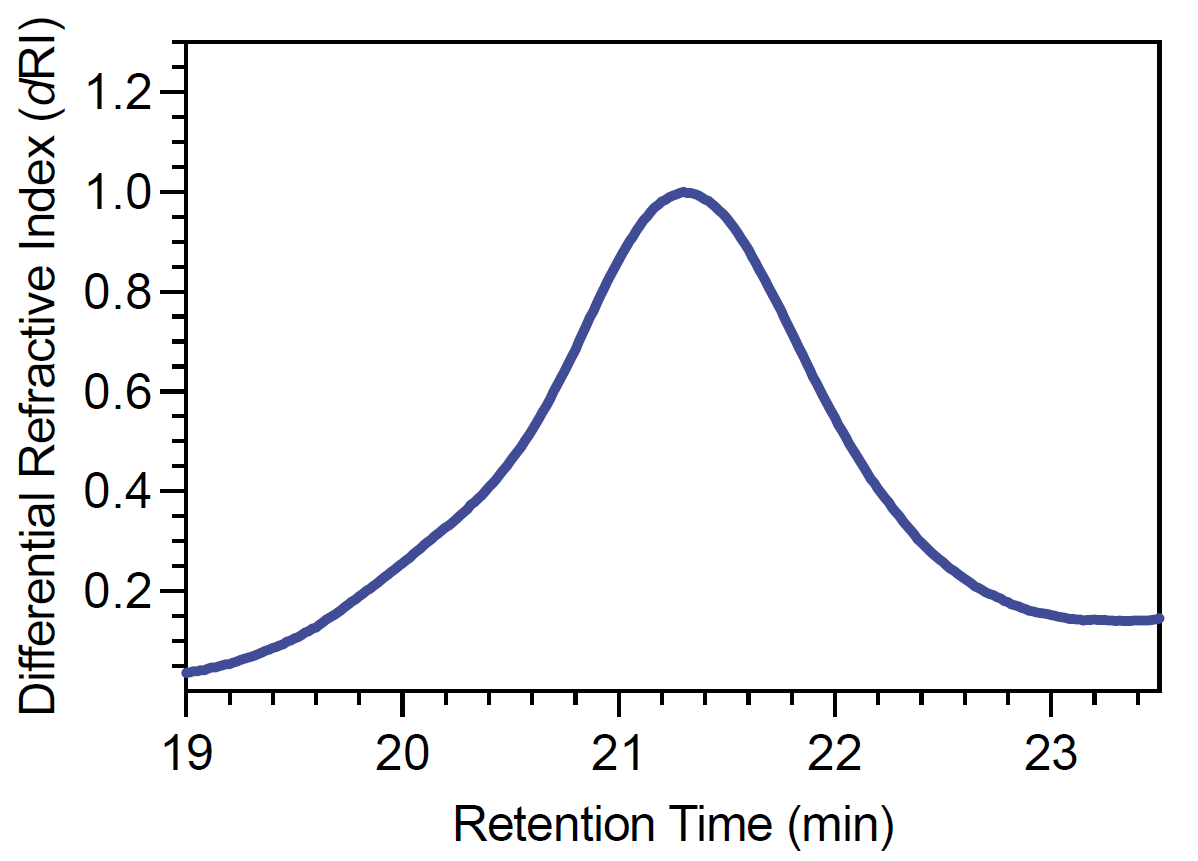


**Figure S1.** *DMF GPC characterization of PEG-b-(PHMA-co-DASA): M_n_ = 18,768 g mol^-1^, Ð = 1.23.*


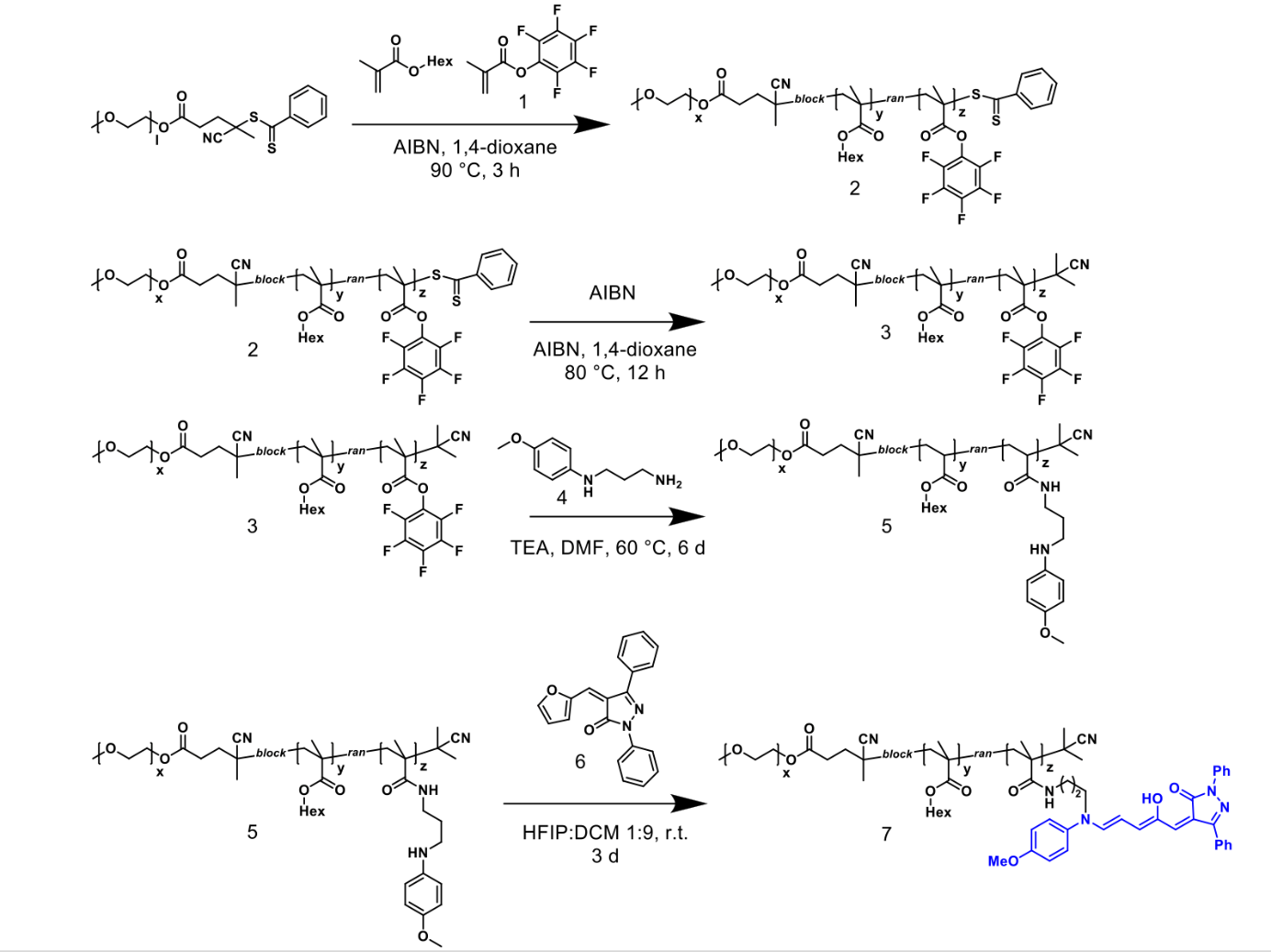


**Figure S2.** *Schematic for synthesis of DASA-conjugated polymer (PEG-b-(PHMA-co-DASA))*


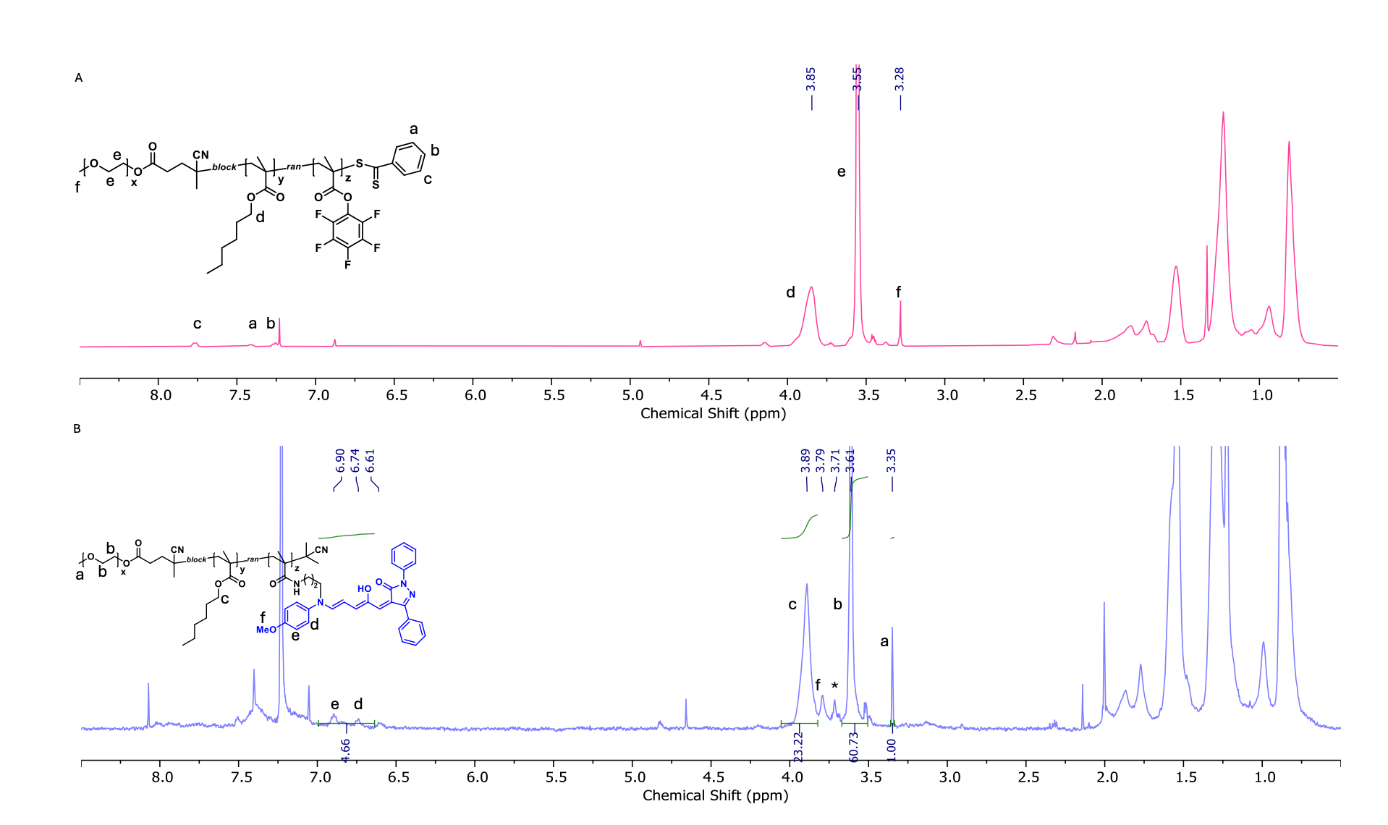
**Figure S3.** *^1^H NMR of the DASA-conjugated polymer.*

**
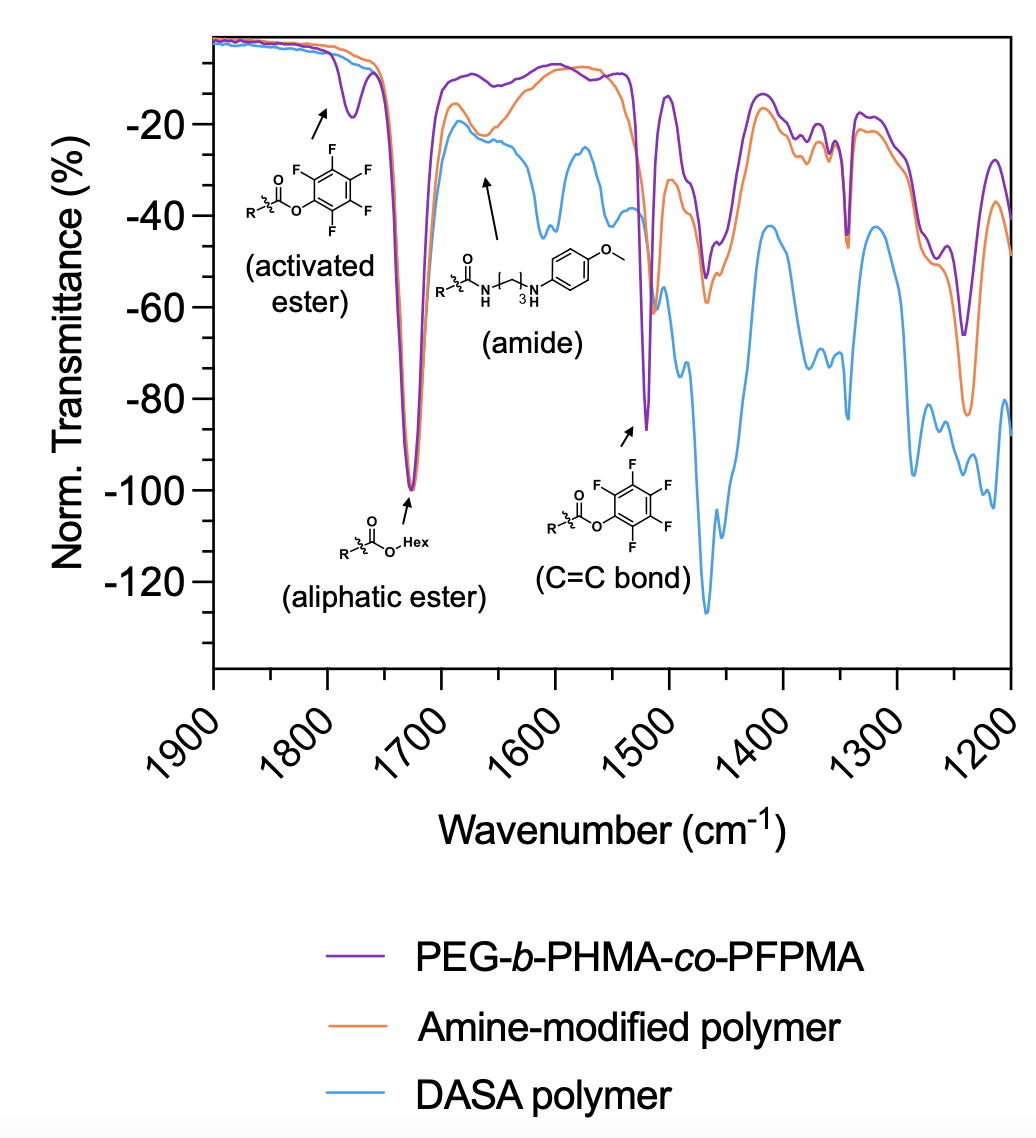
**

**Figure S4.** *FTIR spectra overlay for PEG-b-PHMA-co-PFPMA, amidification via MPDP addition of polymer, and PEG-b-(PHMA-co-DASA).*


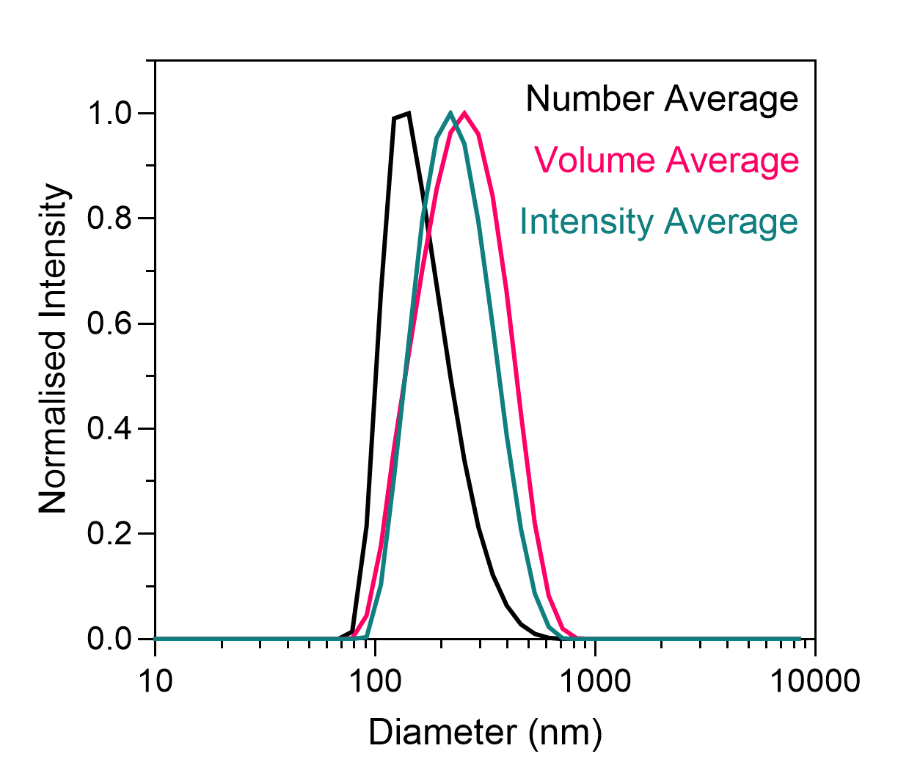


**Figure S5.** *DLS measurement of cargo-free DASA polymersomes showed particle diameters, D_h_* = 217.3 ± 1.4 nm*. These were formed by solvent exchange and purified by dialysis.*

*Characterization of Dye Release from Polymersomes in Aqueous Dispersion*

When samples were kept entirely in the dark, only negligible fluorescein release was observed. Notably, when samples were kept in aluminium foil wrapped vials, and only exposed to low levels of ambient light (for example, when taking aliquots), elevated levels of fluorescein release were recorded. Although this was of a lower magnitude than the fluorescein release which was observed upon continuous irradiation of the polymersomes at 630 nm, this is indicative that the permeability of the polymersomes is sensitive to even low intensities of light exposure. This could indicate that even small shifts in equilibrium between the “open” triene-enol and the “closed” cyclopentenone forms of the PYRA can cause significant payload release. The implication of this is that any implementation of the hydrogels reported herein for a drug-release application would require careful handling of the materials to prevent incidental light exposure. This is an important limitation which would need careful consideration before further translation of this technology. However, this is also a promising finding for other applications. Similar DASA-based light-responsive polymersomes have been reported for use as nanoreactors which can capitulate basic cellular functions such as circadian rhythm.^1^ The use of light as a tool for precise modulation of the equilibrium state of a system is an important consideration in the design of such systems, and hence the increased light-sensitivity indicated herein could lead to more precise stimulation at lower intensities of incident light.


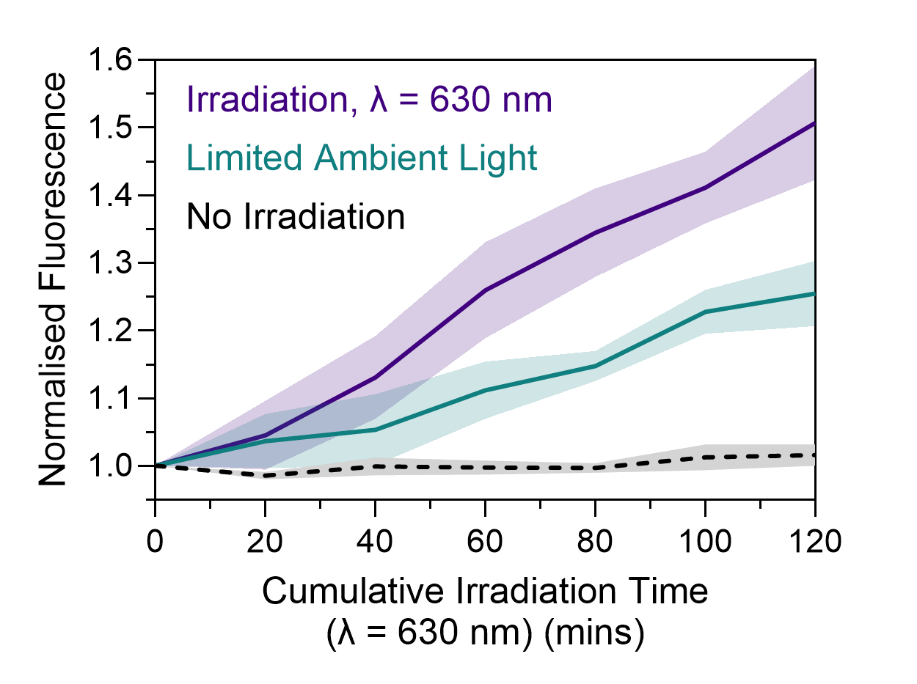


**Figure S6.** To investigate the effect of concentration on the self-assembly process, polymersomes were prepared at 0.1 mg mL^-1^, 0.2 mg mL^-1^ and 0.4 mg mL^-1^ of DASA-polymer in dispersion. This range of concentrations showed good reproducibility (both between replicates and relative to each other), demonstrating that the switching behavior was consistent between the different preparation methods. Moreover, the data provide clear evidence that the photoswitching functionality of the DASA was preserved upon self‑assembly of the amphiphilic block copolymer into polymersomes, showing that it is an appropriate stimuli-responsive functionality for light-initiated cargo release. Limited dye release observed when DASA-polymersomes were exposed to low levels of ambient light while in aqueous dispersion. Fluorescence spectroscopy was used to monitor release of a fluorescein disodium salt cargo from DASA polymersomes in aqueous dispersion. The aqueous polymersome dispersion was irradiated continuously (630 nm), monitoring fluorescence at 20-minute intervals. This was compared to equivalent dispersions exposed to limited ambient light and kept in the dark as a control. (N = 3, SD shown).

*Calculations for Dye Release (%) from Polymersomes*

Following the method set out by Rifaie-Graham *et al.*,^2^ sodium fluorescein release studies were quantified to represent dye release as a percentage of the total loading capacity for each particle batch. This was calculated using a calibration curve for absorbance of fluorescein at varying concentrations to determine the extinction coefficient of sodium fluorescein used in this work. Another calibration curve for fluorescence intensity versus fluorescein concentration gave the slope (m) used to calculate the maximum fluorescence intensity of the particles. Finally, the maximum absorbance (A) at λ = 493 nm was measured for each particle batch, using a 1 cm path length cuvette (b). Using these values, the loading capacity in terms of maximum fluorescence (F_max_) was calculated as shown below:

$$F_{max}\left( a.u. \right)=m\times c_{sodium fluorescein}=m\times\frac{A}{b\times\varepsilon}$$

Using the F_max_ values calculated for each particle batch, dye release (%) was calculated as shown, where F is the maximum fluorescence at 515 nm:

$$F_{\%}\left( \% \right)=\frac{F(a.u.)}{F_{max}(a.u.)}\times100$$

Additionally, encapsulation efficiency was calculated using the following equation:

$$EE\%= \frac{encapsulated cargo (mol)}{total amount of cargo added (mol)}\times100$$

*Characterization of the PEG DBCO Macromer*

Characterization of the 4-arm PEG-DBCO and PEG-azide by ^1^H NMR (D_2_O) gave a degree of functionalization (DoF) of 90% for PEG-azide and 57% for PEG-DBCO. The DoF for the PEG-azide spectrum was calculated from penterythritol singlet reference peaks for the PEG (**1**) and diagnostic end group protons for the DBCO (**4**), labeled on the NMR spectra (**Figure S 4**). The ratio of the integrals of these two peaks gave the DoF, converted to percentage for the PEG to azide groups (**Figure S3**), and repeated for PEG-DBCO (**Figure S4**).

**
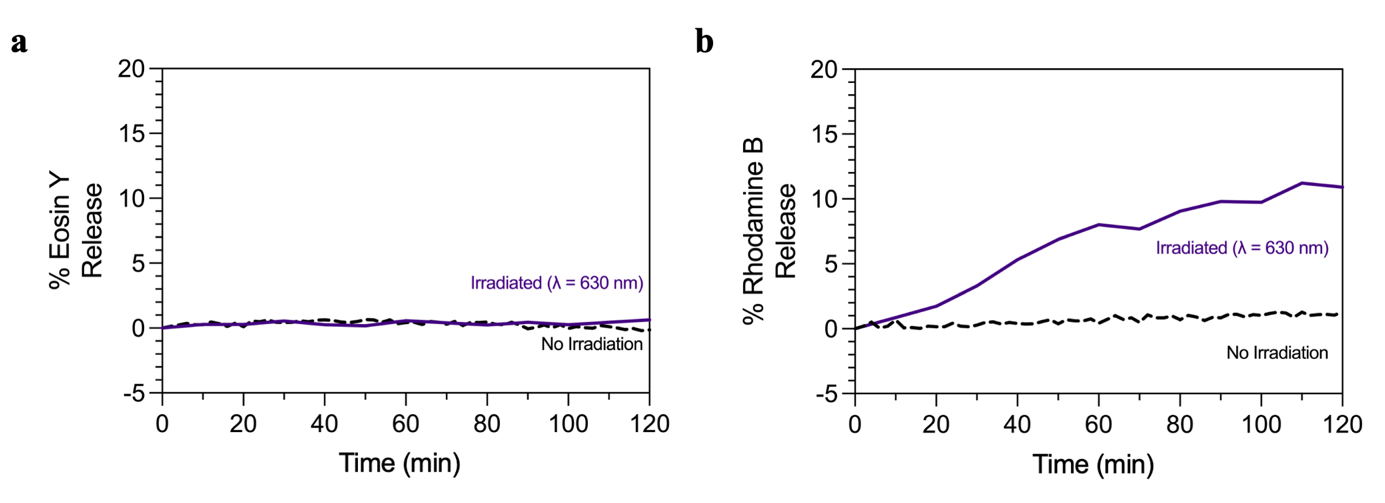
**

**Figure S7.** Release kinetic studies of different small molecule cargos from PEG-*b*-(PHMA-*co*-PDASA) polymersome dispersions in UltraPure water under constant irradiation (λ = 630 nm). Graphs show % dye release, of (a) eosin Y and (b) rhodamine B.

**
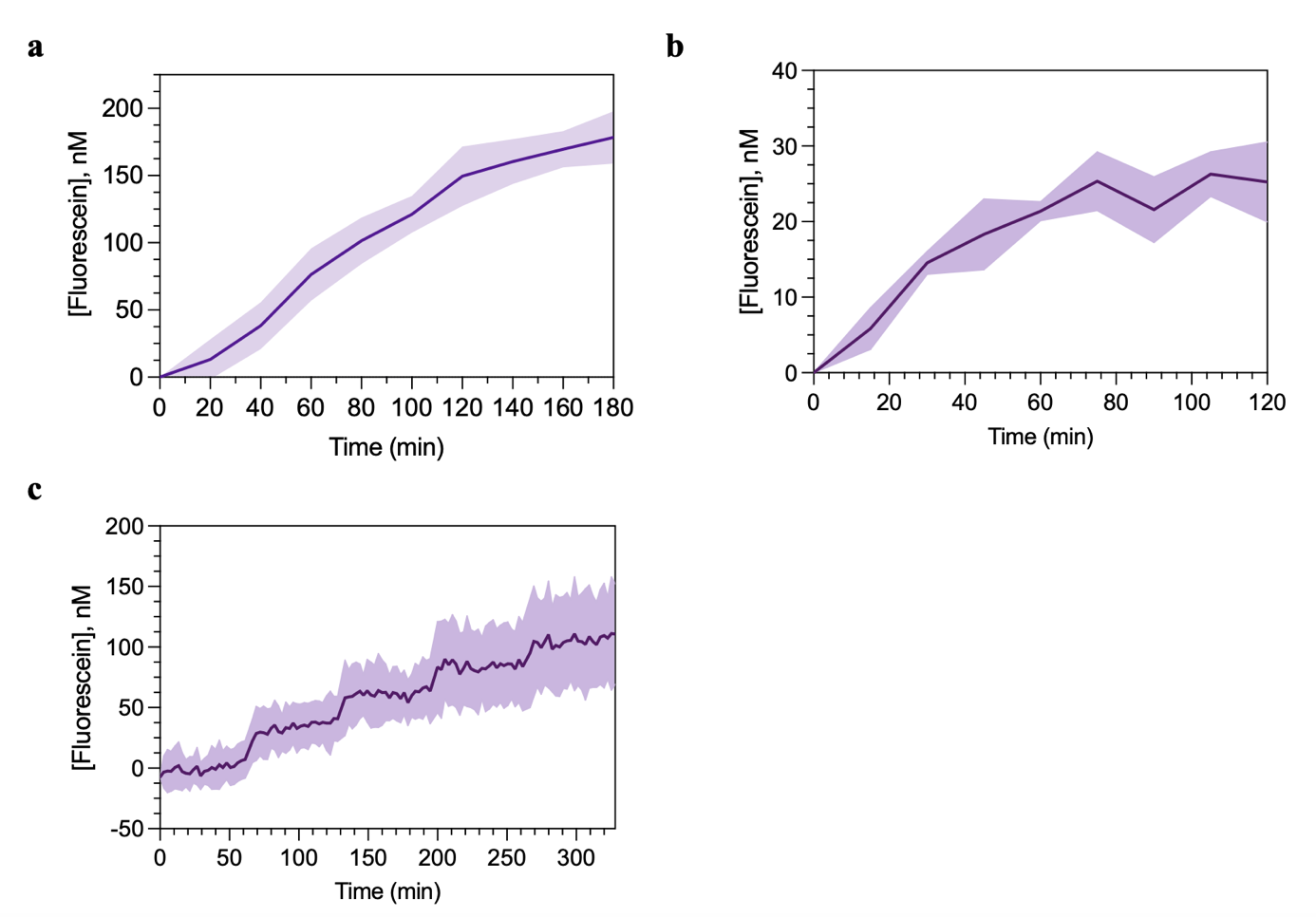
**

**Figure S8.** Fluorescein release quantified, shown as concentration (nM). For **Figure 2D**, particles free in solution, the maximum average concentration reached 178 nM. For **Figure 3C** and **3D**, particles encapsulated within a hydrogel, this reached a maximum average concentration of 26 nM and 111 nM, respectively. (a) Fluorescence spectroscopy was used to monitor release of fluorescein from DASA polymersomes in an aqueous dispersion. The aqueous polymersome dispersion was irradiated continuously (l = 630 nm), monitoring fluorescence at 20‑minute intervals. (b) Fluorescence spectroscopy demonstrating light-induced release of fluorescein from the hydrogel. Hydrogels were irradiated with visible light (λ = 630 nm), and cargo release was monitored at 15-minute intervals over 120 minutes. (c) Cyclic switching experiment to investigate selective control of release rates. Hydrogels were fabricated by a click chemistry reaction between DBCO and azide functionalized PEG macromers in fluorescein-loaded polymersome solution, then irradiated for 5 minutes with visible light (λ = 630 nm). Cargo release was then monitored by fluorescence spectroscopy at regular intervals for 1 hour.

**Figure S9.** *^1^H NMR spectrum for 4-arm PEG-DBCO in D_2_O.*

**

**Figure S10.**  *^1^H NMR spectrum for 4-arm PEG-azide in D_2_O.*


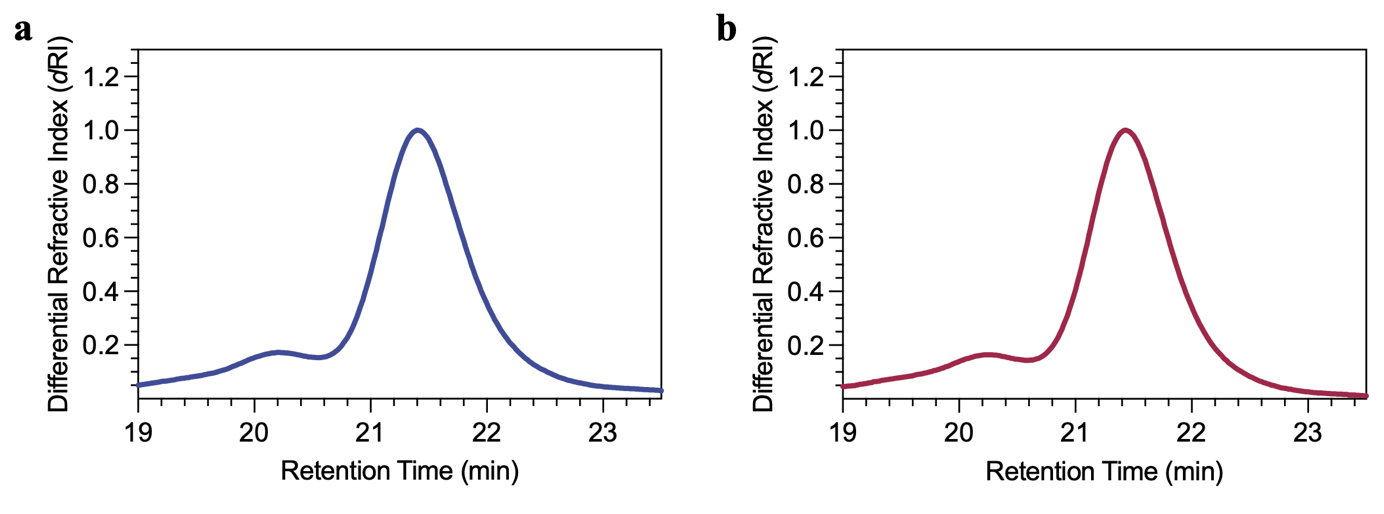
**Figure S11.** *DMF GPC characterization of (a) PEG-DBCO (M_n_ = 18,768 g mol^-1^, Ð = 1.23) and (b) PEG-Azide (M_n_ = 18,613 g mol^-1^, Ð = 1.28).*

**
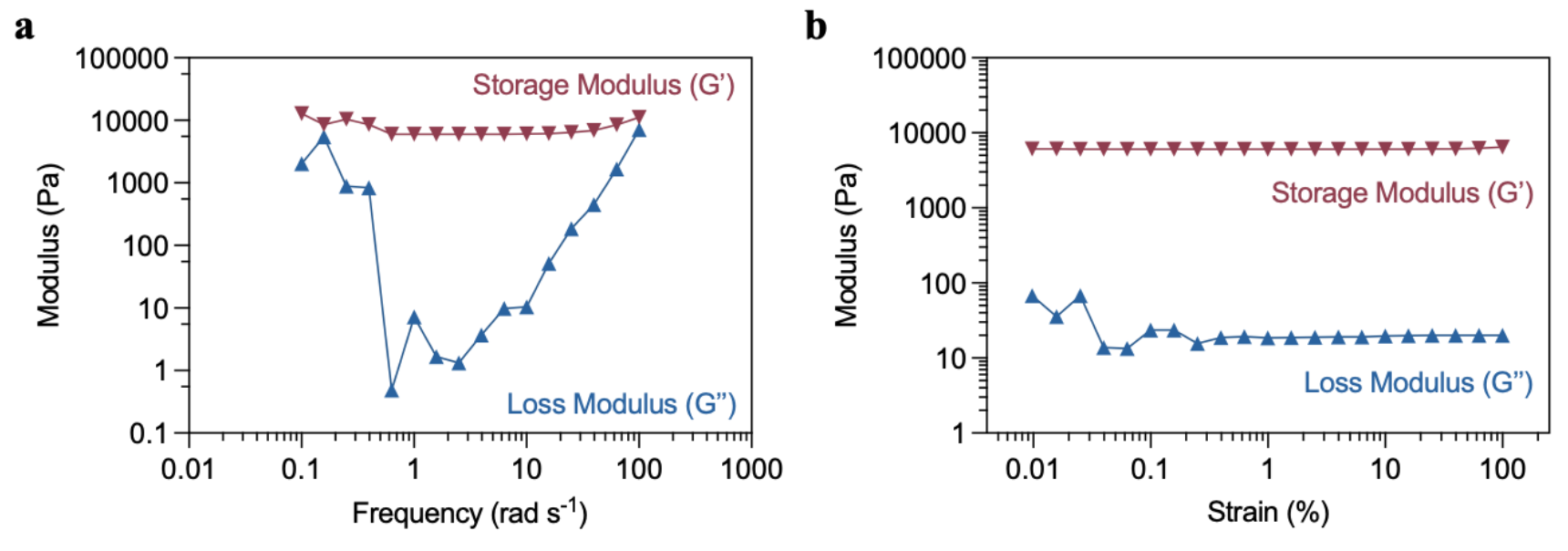
**

**Figure S12.** *Rheology measurements for PEG-azide-DBCO hydrogels (100 mg mL^-1^). Storage and loss modulus of the crosslinked gel under a (a) frequency sweep and (b) strain sweep.*


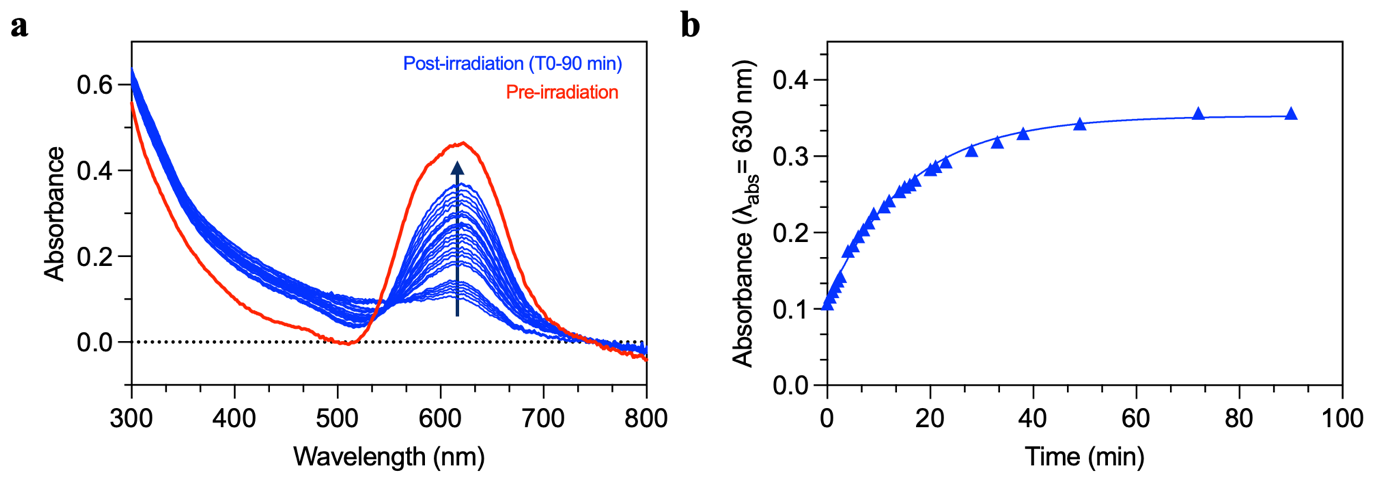
**Figure S13.** *Absorbance Spectra of DASA Polymersomes for Half Life Determination.* (a) Absorbance spectra before irradiation at λ = 630 nm (red) and after irradiation (blue), at regular intervals between T = 0-90 min, with arrow showing change in absorbance during conversion back to the 'open' tautomer. (b) Absorbance as a function of time to determine half-life (t_1/2_ = 10.8 min) using a one-phase association non-linear fit model.

**References**

1. Rifaie-Graham, O. *et al.* Photoswitchable gating of non-equilibrium enzymatic feedback in chemically communicating polymersome nanoreactors. *Nat Chem* **15**, 110–118 (2023).
2. Rifaie-Graham, O. *et al.* Wavelength-Selective Light-Responsive DASA-Functionalized Polymersome Nanoreactors. *J Am Chem Soc* 140, 8027–8036 (2018).
